# The T7-Related *Pseudomonas putida* Phage ϕ15 Displays Virion-Associated Biofilm Degradation Properties

**DOI:** 10.1371/journal.pone.0018597

**Published:** 2011-04-19

**Authors:** Anneleen Cornelissen, Pieter-Jan Ceyssens, Jeroen T'Syen, Helena Van Praet, Jean-Paul Noben, Olga V. Shaburova, Victor N. Krylov, Guido Volckaert, Rob Lavigne

**Affiliations:** 1 Laboratory of Gene Technology, Katholieke Universiteit Leuven, Leuven, Belgium; 2 School of Life Sciences, Biomedical Research Institute and Transnational University Limburg, Hasselt University, Diepenbeek, Belgium; 3 State Institute for Genetics of Industrial Microorganisms, Moscow, Russia; University of Birmingham, United Kingdom

## Abstract

Formation of a protected biofilm environment is recognized as one of the major causes of the increasing antibiotic resistance development and emphasizes the need to develop alternative antibacterial strategies, like phage therapy. This study investigates the in vitro degradation of single-species *Pseudomonas putida* biofilms, PpG1 and RD5PR2, by the novel phage ϕ15, a ‘T7-like virus’ with a virion-associated exopolysaccharide (EPS) depolymerase. Phage ϕ15 forms plaques surrounded by growing opaque halo zones, indicative for EPS degradation, on seven out of 53 *P. putida* strains. The absence of haloes on infection resistant strains suggests that the EPS probably act as a primary bacterial receptor for phage infection. Independent of bacterial strain or biofilm age, a time and dose dependent response of ϕ15-mediated biofilm degradation was observed with generally a maximum biofilm degradation 8 h after addition of the higher phage doses (10^4^ and 10^6^ pfu) and resistance development after 24 h. Biofilm age, an in vivo very variable parameter, reduced markedly phage-mediated degradation of PpG1 biofilms, while degradation of RD5PR2 biofilms and ϕ15 amplification were unaffected. Killing of the planktonic culture occurred in parallel with but was always more pronounced than biofilm degradation, accentuating the need for evaluating phages for therapeutic purposes in biofilm conditions. EPS degrading activity of recombinantly expressed viral tail spike was confirmed by capsule staining. These data suggests that the addition of high initial titers of specifically selected phages with a proper EPS depolymerase are crucial criteria in the development of phage therapy.

## Introduction

Biofilms are surface-associated complex bacterial communities encased in a hydrated extracellular polymer matrix of exopolysaccharides (EPS), proteins, nucleic acids and lipids. They are formed spontaneously on both inert and living systems in various natural and man-made environments, from food processing to industrial (*e.g.* water pipes) and hospital settings (*e.g.* burn wounds, endocarditis and catheters). They represent an essential bacterial survival strategy since biofilm-associated bacteria can reach a thousand fold increased protection against antimicrobial agents compared to their planktonic counterparts [1], [2]. Very often the antibiotic concentration to eradicate the biofilm is above the peak serum concentration, rendering it ineffective in treating biofilm infections [3]. As they also confer protection for host defense mechanisms, biofilms are a leading cause of latent as wells as of recurrent infections [4], [5].

*Pseudomonas putida* belongs to the fluorescent group of the *Pseudomonas* species, a group of opportunistic pathogens that primarily cause nosocomial infections. In contrast to *Pseudomonas aeruginosa*, the most prevalent pathogen, infections caused by *P. putida* are rare and mostly reported in immunocompromised patients, such as newborns [6], neutropenic and cancer patients [7]–[9]. The ability to adhere to materials and to promote the formation of biofilms appears the most important feature of the pathogenicity of *P. putida* [10]. Despite a high susceptibility for anti-pseudomonal β-lactams, multidrug-resistant *P. putida* isolates to β-lactams, including carbapenems, have already been reported [11]–[15].

The widespread emergence of resistance to antibiotics among pathogenic bacteria emphasizes the need to explore new classes of antibacterial agents, ones that cannot be resisted by the same antibiotic resistant genes. (Bacterio)phage therapy (use of bacterial viruses) may represent such a new class, with additional advantages like self-replication at the site of infection and host specificity, leaving the normal bacterial flora undisturbed [16]. To penetrate EPS layers, some phages carry EPS depolymerases as tail spikes or tail fibers, as part of the viral particle, to enable them to reach the bacterial cell wall [17]. Consequently, phages cause biofilm and capsule disruption by cell infection and lysis, as well as by EPS degradation. The principle of EPS depolymerization as biofilm destabilizing agent was first employed in the pre-antibiotic era: potentially lethal pneumococcal infections in rabbits and monkeys were controlled through administration of partially purified depolymerase [18], [19].

Despite biomedical and industrial interest for phages encoding these enzymes, recent more in-depth research is limited and mainly focused on the capsulated neuroinvasive *Escherichia coli* K1 strains [20], [21]. Although relatively large numbers of pseudomonad phages have been found in many of the environments in which biofilms are formed, and despite the increasing volume of studies on pseudomonad biofilms, only few groups have examined the effect on biofilms of pseudomonad phages with associated EPS depolymerases. Besides three historical references from Bartell and colleagues [22]–[24], only two recent descriptive studies have been published on the isolation of phages capable of degrading the extracellular material elaborated by its *P. putida* or mucoid *P. aeruginosa* hosts [25], [26].

The present study investigates the *in vitro* biofilm degradation capacity of a lytic *P. putida* phage ϕ15 with associated EPS depolymerase on different aged single-species biofilms of two *P. putida* strains, PpG1 and RD5PR2. In addition, we identified the gene encoding for the EPS depolymerase within the sequenced phage genome.

## Materials and Methods

### Phage, bacterial strains and media

Phage ϕ15 and *P. putida* strains (PpN, PpN3, PpN119, Pput 373, AC522, DSM9278 and PpG1) were isolated as described in Shaburova *et al.* [25]. PpKT2440 and Pp00S82 were kindly provided by Prof. D. Springael (Division Soil and Water Management, K.U.Leuven, Belgium) and five other *P. putida* strains (PSE072, PSE072B, PSE106, PSE107 and WGE060) were obtained from Prof. M. Vaneechoutte (Laboratory Bacteriology, Ghent University Hospital, Belgium). Prof. R. De Mot (Centre of Microbial and Plant Genetics, K.U.Leuven, Belgium) provided *P. putida* strains ATCC23287, GR12-2R3, LMG2257 and a selection of 35 *P. putida* rice rizosphere strains [27]. *P. putida* strains were grown in standard LB-medium at 30°C. Expression strain *E. coli* BL21(DE3) pLysS (Invitrogen Corporation, Carlsbad, CA, USA) was grown at 30°C in 2xTY-medium.

### Phage purification

The CsCl ultracentrifugation method was performed as described by Ceyssens *et al.* [28]. For purification and concentration by Convective Interactive Media (CIM), the diethylamine (DEAE) CIM® disk monolithic column (12 mm×3 mm i.d., bed volume 0.34 ml; BIA Separations, Ljubljana, Slovenia) was used in combination with the Äkta FPLC-system (GE Healthcare, Little Chalfont, UK) and UNICORN™ 5.01 software in a 20 mM Tris buffer system at pH 7.5. The equilibration buffer with 1 M added sodium chloride was used for elution. Shortly, an overnight culture of a susceptible host strain was used to prepare a 2% inoculum, which was incubated with shaking at 30°C. At OD_600 nm_ of 0.45, phage (10^6^ pfu to a 50 ml culture) were added to the culture and incubation continued until lysis was observed. The bacterial lysate was subsequently centrifuged (3,000× *g*, 30 min, 4°C), passed through a 0.45 µm pore-size membrane filter (Millipore Corporation, Billerica, MA, USA) and adjusted (with HCl or NaOH) to the pH of the buffer system. This cleared, filtered lysate was used as the starting material for phage purification by CIM. In a stepwise gradient profile (20, 30, 100% and 3 ml segment lengths) to the Tris buffer containing 1 M NaCl, the major part of the bound phage were eluted at 30% elution buffer. The DEAE disk, with a capacity of ∼8*10^10^ pfu for phage ϕ15 can purify up to ∼7*10^10^ phage particles.

### Microbiological characteristics

One-step growth curves of phage ϕ15 were made for two *P. putida* strains, PpG1 and RD5PR2 [29]. In short, 5 min after infection of a 1 ml *P. putida* culture (OD_600 nm_ = 0.45) at a start pfu/CFU ratio of 0.008, the suspension was washed (1 min centrifugation at 16.060× *g*, followed by resuspension of the pellet in 1 ml LB, prewarmed at 30°C) twice and 10^−4^ and 10^−5^ dilutions were incubated at 30°C. Samples were taken at regular intervals and titrated immediately using the double agar overlay method. For the adsorption experiments [29], infection parameters of the one-step growth experiments were maintained. At 1 min-time intervals, 100 µl samples were taken from the solution and mixed with 850 µl LB-media and 50 µl CHCl_3_. After 10 min of shaking, the supernatant is titrated to determine the number of non-adsorbed or reversibly adsorbed phage.

The pH-stability of the phage was tested by incubating at room temperature 10^8^ pfu in 1 ml of a pH-buffer constituting of 10 mM KH_2_PO_4_, 10 mM NaCitrate, 10 mM Boric Acid and 150 mM KCl (pH-adjusted with NaOH or HCl to a pH within the pH-range of 1 to 13). Each sample was titrated after a 24 h exposure using the standard double agar overlay method [29], and compared with control samples [10^8^ pfu in a 1 ml standard phage buffer (10 mM MgSO_4_, 150 mM NaCl, 10 mM Tris pH 7.5) also incubated at room temperature for 24 h].

All phage-host combinations were evaluated by spot testing 10^7^ pfu on a air-dried bacterial lawn (200 µl overnight culture plated directly on LB agar plates) in three independent experiments. After overnight incubation at 30°C, plates were checked for the presence of a lysis zone against a negative, uninfected control.

To visualize halo formation, 10 µl of a 10^8^ pfu phage suspension was dropped on a air-dried bacterial lawn (200 µl overnight culture), plated on LB agar. Plates were sealed and incubated at 30°C. For comparison of phage and bacterial count in the three different zones (lysis, halo and bacterial zone), a equally large surface was removed from each zone, suspended in 1 ml LB-medium and vortexed (30 s). Phage counts were determined using the double agar method, while bacterial numbers were counted after plating a dilution series of the supernatant.

### Development of phage resistant strains and UV inactivation of phage particles

200 µl of a bacterial overnight culture was plated on a petri dish with solid LB medium. After drying, 10 µl of 10^8^ pfu/ml of a ϕ15 phage suspension was dropped on this plate, which was sealed after drying and incubated at 30°C until colonies became visible in the lysis zone. Colonies were picked and subjected to single-colony isolation three times. The double agar overlay method was used to verify resistance to phage ϕ15 of the different selected colonies.

The UV oven (Bio-Link, Vilber Lourmat, France) was equipped with five fluorescent lamps of 8 W each, emitting 180 to 280 nm with a peak at 254 nm. The distance between the lamps and the plates was 14 cm. The UV doses were programmed and controlled by a radiometer which constantly monitors the UV light emission. To inactivate phage particles by UV illumination, 25 µl of phage (10^11^ pfu/ml) was dropped on an empty petri dish and irradiated in the UV oven (1 kJ/m^2^).

### Capsule staining

Bacterial cells, scraped from a fully grown agar plate, were transferred to a 10 µl drop of 1% aqueous Congo red solution (Sigma-Aldrich, St. Louis, MO, USA). This suspension was spread across a microscopic glass slide to form a thin film, which was air-dried. A 10 µl drop of Maneval's solution [3.33% fenol, 4.44% glacial acetic acid, 2.67% ferric chloride, 0.02% acid fuchsin (Sigma-Aldrich)], distributed across the slide, acidifies the Congo red background into blue [30] and colors cells red. Capsules are not stained and appear white underneath the light-microscope (100×, oil; Leica DM LB microscope; Mc Bain instruments, Simi Valley, CA, USA).

### Biofilm assay

The device used for biofilm formation is a platform of 96 polystyrene pegs (Nunc-Immuno TSP, Thermo Fischer Scientific, Tournai, Belgium) that fits as a microtiter plate lid with a peg hanging in each microtiter plate well (Thermo Fischer Scientific) [31]. Overnight cultures were diluted 1/200 in LB-medium in the 96 wells of the microtiter plate. After hanging the pegs in the wells, the microtiter plate was sealed to avoid water loss due to evaporation. Biofilms were allowed to grow for different periods of times at 30°C without shaking. For biofilm growth assays longer than 24 h, media were renewed every 24 h by placing the pegs in a new microtiter plate containing media. For quantitative biofilm analysis, the pegs were washed once in 200 µl LB-medium. The remaining attached bacteria were stained with 200 µl 0.1% (w/v) crystal violet (Merck) in an isopropanol-methanol-PBS solution [1∶1∶18 (v/v)] for 30 min, washed with LB-medium to remove excess stain and air-dried (30 min). Afterwards, the dye bound to the adhered cells was extracted in 200 µl 30% glacial acetic acid. The intensity of the eluted dye in each well was measured at OD_600 nm_ with the Multiskan RC (Thermo Labsystems, Finland). The planktonic survival was determined simultaneously by an optical density measurement (600 nm; Multiskan RC) of 135 µl supernatant removed from the microtiter plate in which the pegs were hanging. Differences in biofilm and planktonic survival compared to the untreated control samples were analyzed using the two-tailed *t* test, P-values of <0.01 were considered significant.

### Cloning, recombinant expression and purification

Purified genomic ϕ15 DNA served as template for amplification of gene *17* using Pfu DNA polymerase (Fermentas Life Science) and the specific primer pair (5′-ATGGCACGAACTATCGTC-3′ and 5′-CTACCCGACCAGCTCGATCAG-3′; Eurogentec, Seraing, Belgium). The PCR product was cloned in the pEXP5-NT/TOPO® TA expression vector (Invitrogen Corporation) according to the manufacturer's protocol in order to obtain a 6xHis-tag fusion protein for purification. The construct was verified by DNA sequencing using vector-specific primers (5′-TAATACGACTCACTATAGGG-3′ and 5′-TAGTTATTGCTCAGCGGTGG-3′, Eurogentec) and an internal primer (5′-ACGTGGTGTCATCAAC-3′, Eurogentec). Expression occurred at 37°C for 4 h in *E. coli* BL21(DE3)pLysS (Invitrogen Corporation) after induction with 0.1 mM isopropyl-β-D-thiogalactopyranoside of 0.5 L exponentially growing cells (OD_600 nm_ = 0.6) in 2xTY medium. Protein purification was essentially performed as described previously [32] with a HisTrap™ HP 1 ml column (Amersham Biosciences) in combination with an Äkta FPLC-system (GE Healthcare) and UNICORN™ 5.01 software. Wash and elution buffers were composed of 20 mM NaH_2_PO_4_ pH 8.5, 0.5 M NaCl and 10% glycerol with 75 mM and 0.5 M imidazole, respectively. Protein purity was at least 95% as estimated by SDS-PAGE. Purified protein was dialyzed overnight against a volume of PBS buffer (137 mM NaCl, 2.7 mM KCl, 10 mM NaHPO_4_, 2 mM KH_2_PO_4_ pH 7.4) that was 1000× the volume of the protein sample using Slide-A-Lyzer® MINI Dialysis units (Pierce Biotechnology, Rockford, IL, USA).

## Results

### Microbiological characteristics

Bacteriophage ϕ15 propagates on *P. putida* strain PpG1 and infects six other strains (PpN, PpN3, RD6PR1, RD5PR2, RD8PR2 and RD8PR3) from a diverse collection of 53 *P. putida* strains. These seven *P. putida* strains show very diverse biofilm forming capacities, ranging from a very weak to a moderate biofilm formation (Figure S1). None of the phage-sensitive strains forms thick biofilms.

Transmission electron microscopic imaging revealed ϕ15 as a typical short tailed member of the *Podoviridae* family of double-stranded DNA bacteriophages (58.9 nm head; 12×8 nm tails) (Figure S2). Phage ϕ15 is stable in a relatively narrow pH range (5 to 11) (Figure S3A). The phage exhibits a very efficient infection cycle on its host PpG1: 95.2% of ϕ15 phage particles are irreversible adsorbed (k = 2.51*10^−8^) within the first minute after infection of a bacterial culture (Figure S3B). PpG1 host cells are lysed after 21 min, releasing an average of 95 newly formed phage particles per cell (Figure S3C). However, under the same laboratory conditions, ϕ15 exhibited a remarkably lower adsorption to strain RD5PR2 (17.38% adsorbed phage particles after 1 min; k = 2.23*10^−9^) (Figure S3B). As a consequence, lysis of RD5PR2 cells occurred unsynchronized, which was observed by a gradual release of phage particles starting 90 min post infection (Figure S3D).

**Figure 1:**
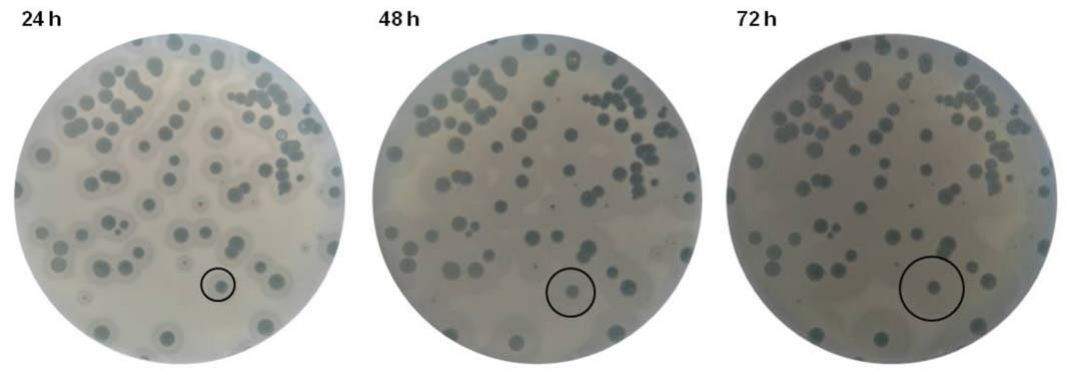
Plaques of *P. putida* phage ϕ15 on the host PpG1. With increasing time of incubation the diameter of the halo zone, surrounding the plaque with constant diameter, increases up to 3 mm every 24 h.

The large (5-mm diameter) and clear plaque morphology of ϕ15 hints a strictly lytic nature. The most distinct plaque feature is the opaque halo zone with an increasing diameter over the course of time, surrounding a plaque with constant diameter (Figure 1). When 10^8^ pfu of phage were dropped on a lawn of host bacteria, the lysis zone caused by infection and subsequent lysis of host cells, kept a constant diameter with prolonged incubation. The halo zone increased up to 3 mm in diameter every 24 h and eventually spread across the whole petri dish. An opaque halo zone surrounding the plaque was observed for each of the seven strains (PpG1, PpN, PpN3, RD6PR1, RD5PR2, RD8PR2, RD8PR3) out of the collection of 53 *P. putida* strains sensitive for ϕ15 infection. A halo zone was not observed when phage were dropped on a bacterial lawn resistant to infection, coupling phage sensitivity and halo formation. Phage enumeration of equally large surfaces of the lysis, halo and bacterial zones outside the halo revealed that only a tenfold less phage were found in the halo zone (1.6*10^6^ pfu) compared to the lysis zone (1.9*10^7^ pfu), while phage were completely absent in the outside bacterial zone. A bacterial count of the same samples indicated the absence of viable bacterial cells within the lysis zone, except for the one or two resistant colonies. The halo and the outside bacterial zone harbored an almost equal amount of viable cells (4.56*10^7^ and 4.43*10^7^ CFU, respectively).

### Phage-mediated biofilm degradation

Phage-mediated biofilm degradation was evaluated for phage ϕ15 on 24 h and 48 h old single-species biofilms of two *P. putida* strains PpG1 and RD5PR2, grown under static conditions at 30°C. Infection parameters were varied to determine the most optimal biofilm degradation conditions: time periods for phage infection ranged from 2 to 24 h and the applied phage titers were 10^2^, 10^4^ and 10^6^ pfu/well.

#### Biofilm degradation of PpG1 biofilms

**Figure 2:**
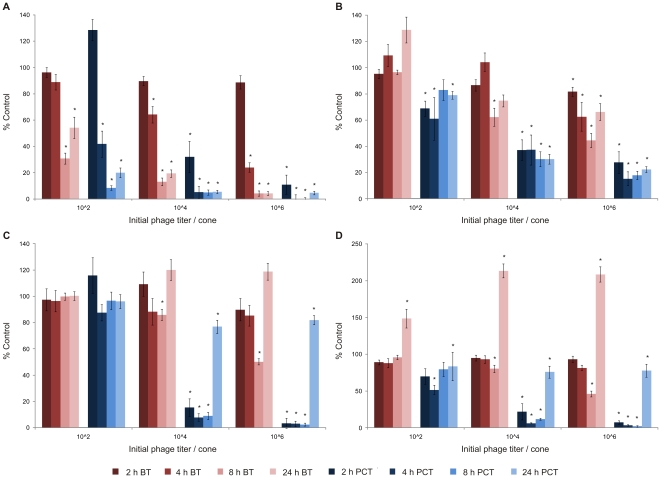
Phage ϕ15-mediated biofilm degradation. 24 h (A) and 48 h (B) old biofilm of PpG1. 24 h (C) and 48 h (D) old biofilm of RD5PR2. Biofilms grown on cones for 24 or 48 h, were initially inoculated with 10^2^, 10^4^ and 10^6^ pfu of phage ϕ15. After 2, 4, 8 and 24 h of phage treatment at 30°C, mean biofilm (BT: biofilm treatment) and planktonic survival (PCT: planktonic culture treatment) for n = 20 cones or corresponding microtiter plate wells, respectively, were scored relative to untreated control samples (100%) and represented on the Y-axes. Five independent experiments were performed, each starting from a different overnight culture and each with four repeats for each parameter combination giving rise to an overall twentyfold repetition. Error bars indicate SEM. Significant values (two-tailed *t* test; P<0.01) are marked by a asterisk (*).

A time and dose dependent biofilm degradation is observed for ϕ15 infection of PpG1 biofilms initially grown for 24 h. A maximal biofilm reduction was reached 8 h after each phage dose was added (*e.g.* 96% for initially 10^6^ pfu added) (Figure 2A). The planktonic survival decreased in parallel, but more rapidly than the biofilm degradation. Only 2 h after phage exposure, the planktonic survival decreased to 32 and 11% for 10^4^ and 10^6^ pfu of ϕ15, respectively. The maximum of planktonic decrease was reached already 4 h after addition of the higher phage doses (10^4^ and 10^6^ pfu) and after 8 h for initially 10^2^ phage particles added.

**Figure 3:**
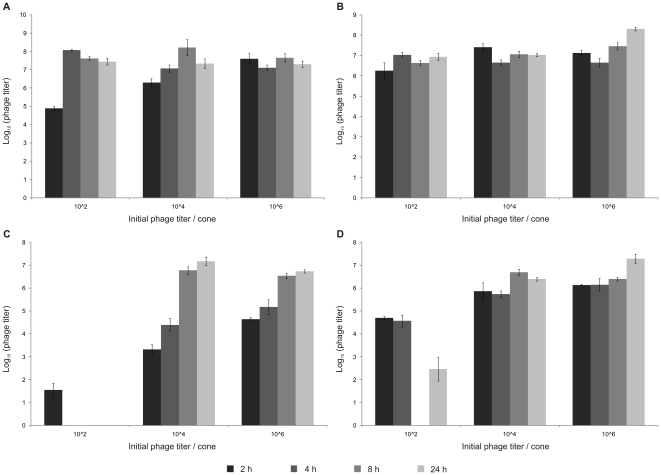
Phage amplification. 24 h (A) and 48 h (B) old biofilm of PpG1. 24 h (C) and 48 h (D) old biofilm of RD5PR2. Biofilms grown on cones for 24 or 48 h, were initially inoculated with 10^2^, 10^4^ and 10^6^ pfu of phage ϕ15. After 2, 4, 8 and 24 h of phage treatment with 10^2^, 10^4^ and 10^6^ pfu of phage ϕ15 at 30°C, mean phage counts recovered from liquid media in n = 4 microtiter plate wells [log_10_(phage titer)] were scored. Error bars indicate SEM.

Despite this dose and time responsive behavior, phage counts within the planktonic culture rapidly attained the steady state level of about 7.5 log_10_ for every initial phage titer added (Figure 3A). Even for only a hundred phage particles added, the maximum phage count (±7.5 log_10_) was already reached after 4 h, at which point also a sharp decrease in planktonic survival (from 128 to 42%) was observed. Surprisingly, biofilm degradation lagged at least 2 h behind, as a sharp increase in biofilm degradation was only observed between 4 and 8 h (11 to 69%) after initial infection. Similar observations were made for higher initial phage titers: the plateau level of phage count was already reached after 2 h at which point the biofilm degradation was still limited to ±11%. A possible explanation is that free living planktonic cells are easier accessible for phage infection than cells associated within and protected by a biofilm environment. Cells within a biofilm environment are also much less metabolic active, hereby slowing down the infection process, and coupled biofilm destruction.

Although also being time and dose dependent, at every time point the measured biofilm degradation and planktonic killing of the 48 h old biofilm were much lower than for the 24 h old biofilm (Figure 2A+B). Like for the 24 h old biofilm, a maximal degradation was reached for the higher phage doses after 8 h, but only 38 and 55% of the biofilm was degraded compared to 87 and 96% for the 24 h old biofilm after initial inoculation with 10^4^ and 10^6^ phage, respectively. When the 48 h grown biofilm was inoculated with only 10^2^ pfu of ϕ15, no significant biofilm degradation was observed during the 24 h incubation period, in contrast with a maximal biofilm degradation of 69% reached after 8 h for the 24 h old biofilm. In parallel, we noticed higher planktonic survival rates for 48 h old biofilms: as these older biofilms slowly decay, biofilm-freed cells hereby continuously enrich the planktonic culture. Nevertheless, phage amplification occurred as rapidly as observed for the 24 h old biofilm, reaching steady-state values of about 7.5 log_10_ at the earlier time points for every initial phage titer applied (Figure 3A+B). A gradual biofilm and planktonic regrowth was observed 24 h after phage exposure for the different phage inoculation titers (except for 10^6^ phage added to the 24 h old biofilm) for both the 24 h and 48 h pregrown biofilm implying bacterial resistance development against phage infection.

#### Comparison of biofilm degradation of different *P. putida* strains

Although PpG1 (0.69) forms thicker biofilms than RD5PR2 (0.54), 24 h old RD5PR2 biofilms are less susceptible for biofilm degradation than PpG1 (Figure 2A+C). Only 8 h after addition of at least 10^4^ phage, a first significant degradation was seen. Almost identical observations were made for the 48 h old biofilm of RD5PR2 as for the 24 h old (Figure 2C+D), indicating no difference in biofilm susceptibility between different aged biofilms of this strain.

In contrast to this lower biofilm susceptibility, RD5PR2 planktonic cultures were rapidly lysed in the presence of 10^4^ and 10^6^ pfu of ϕ15. However, the addition of the lowest titer (10^2^ pfu) to the 24 old biofilm did only induce a minor decrease in planktonic survival (maximum of 13% for RD5PR2 compared with 92% for PpG1). Also, no losses were noted in susceptibility of the RD5PR2 planktonic cultures with increasing biofilm age (Figure 2C+D).

24 h after phage inoculation, we observed for both RD5PR2 biofilms a drastic increase in biofilm and planktonic growth: biofilms grew thicker than the wildtype (>100%) and planktonic cultures were regrown to about 80% of the wildtype. As a regrowth is indicative for the development of phage resistant bacteria, this is in sharp contrast with the further increasing phage count (Figure 3C+D). An inefficient adsorption and release of phage particles during RD5PR2 infection can probably explain the further increasing phage titer at a moment of pronounced resistance development.

### Identification of the ϕ15 tail spike protein by genome analysis

Genome sequencing of ϕ15 revealed a typical T7-like genome, comprising 39,562 bp and bracketed by 264 bp direct terminal repeats. The genome contains 50 predicted ORFs, all orientated in the same direction and leaving only 6.3% of the genome noncoding. It can be functionally divided into three regions involved in (i) host conversion, (ii) nucleotide metabolism and DNA replication, and (iii) morphogenesis and host cell lysis (Figure S4 and Table S1). With a overall G+C average of 58.2%, the highest of all ‘T7-like viruses’, ϕ15 approaches the high G+C content (61%) of its host. No tRNA genes were predicted, as expected for a member of the ‘T7-like viruses’.

The entire ϕ15 genome has 55.5% overall DNA similarity to its closest T7-homolog, the *P. putida* phage gh-1 [33] and shares 34 out of 50 ϕ15 ORFs with this phage. While 28 ORFs are typical for ‘T7-like viruses’, six ORFs are unique to ϕ15 and gh-1 and probably result from adaptation to the *P. putida* host. Five of these genes are encoded in the DNA replication region, while the particle structure genes are highly conserved. Fifteen other ORFs (except *ϕ15/16*), unique for ϕ15 and located in the early and middle region, probably represent a further adaptation to a particular group of *P. putida* strains. Using ESI-MS/MS analysis on purified and denatured phage particles, fourteen structural phage proteins were identified (Table S2). This analysis delineated the structural region from *6.7* to *17* and identified also two early and two middle gene products as being part of the structural phage particle. A detailed discussion of the ϕ15 genome is provided in the supplementary data (Text S1, Table S3, Table S4, Figure S5). Based on the genome sequence, a T7-like infection cycle can be predicted for ϕ15.

### Tail spike protein

**Figure 4:**
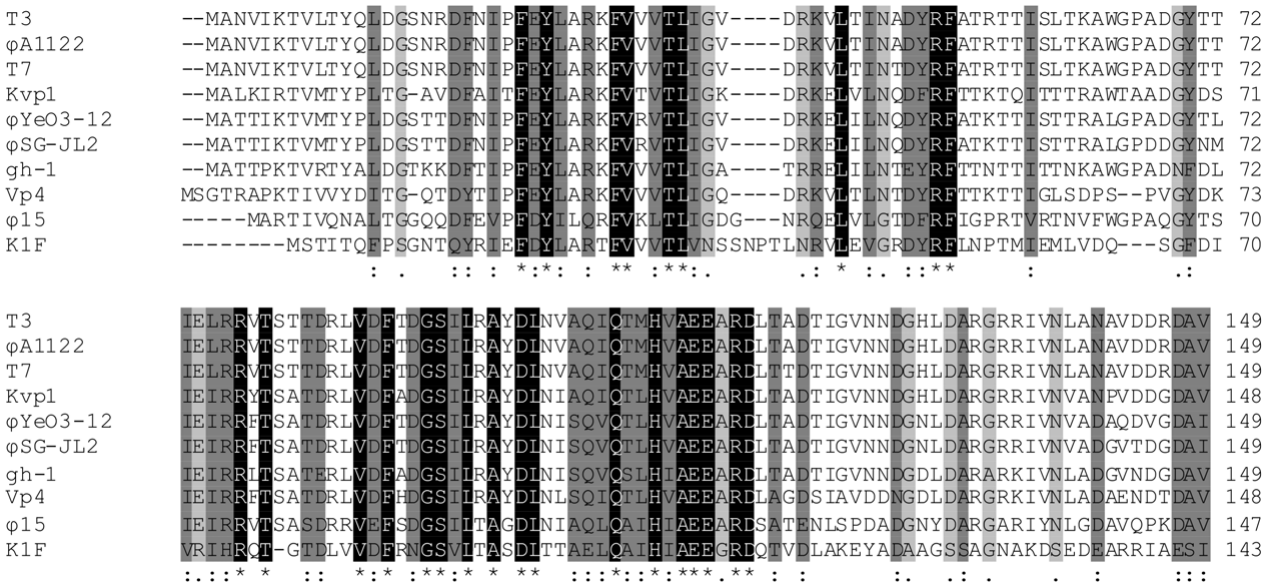
ClustalW alignment of the conserved N-terminal domain of the tail spike proteins (Gp17) of all classified ‘T7-like viruses’. Identical residues are marked in black and similar residues are shaded in two different gray scales, the darker gray marking the higher chemical and physical similarity. Numbering starts at the N-terminal methionine. Gaps, indicated by horizontal lines, were introduced into the sequences to maximize the alignment.

For most ‘T7-like viruses’ (T3, T7, ϕA1122, ϕYeO3-12 and gh-1), the viral tail spike (Gp17) recognizes and adsorbs to specific sugars of the host lipopolysaccharide (LPS) [34], [35]. For example, ϕYeO3-12 is specific for the O3 antigen of *Yersinia enterocolitica*, T3 binds to glucose residues in the outer core of rough *E. coli* strains, while T7 binds to more inner moieties. In contrast, *E. coli* K1 phage K1F encodes a tail-associated endosialidase which recognizes and depolymerizes the K1 polysialic acid capsule instead of LPS structures [36]. Within the classified ‘T7-like viruses’, a conserved N-terminal head-binding domain is present, which is reasonably similar in length (±149 AA) and necessary for association with the tail structure (Figure 4) [37]. Gp17 of ϕ15 (728 AA) shares a 49.7% amino-acid identity in this N-terminal region with gh-1, while the C-terminal two-third of the tail spike is only 18.3% homologous. Also for other members of the ‘T7-like viruses’, these C-termini differ markedly in amino-acid sequence and length. They form the distal part of the tail spike protein and are involved in recognition of and binding to the host cell receptor.

**Figure 5:**
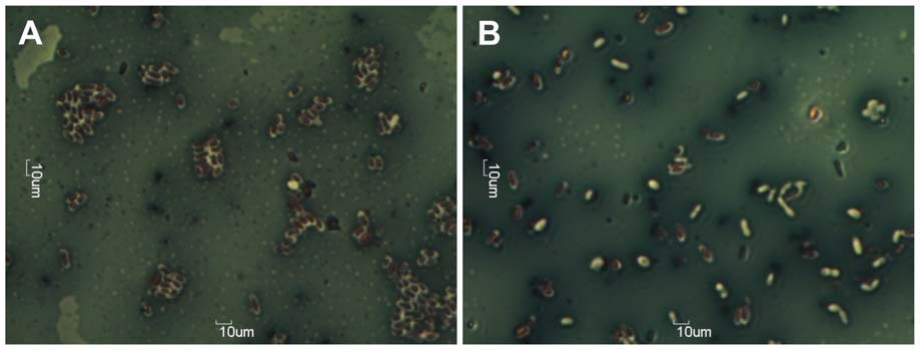
Capsule staining of bacteria in the outside bacterial (A) and in the halo (B) zone. Within the outside bacterial zone bacteria are all encircled by white capsule and packed closely together, while in the halo zone bacteria are lying separately and have sometimes lost their white capsule.

It was expected that the ϕ15 tail spike (Gp17) serves EPS degrading activity. However, homology on the level of the primary amino-acid sequence (BlastP) is limited to the conserved N-terminal domain of the ‘T7-like viruses’. Further analysis of Gp17 of ϕ15 using Phyre [38] indicated that there is a significant chance (75%) that Gp17 of ϕ15 has right-handed β-helical folds, similar to pectin lyases (E-value 0.77). These right-handed β-helical structural elements were so far only identified in carbohydrate binding (and depolymerizing) enzymes of microbial or viral origin and in an autotransporter family of secreted bacterial proteins [39]. Large-scale recombinant expression of the 80 kDa ϕ15 tail spike protein allowed purification of low yet pure amounts of this protein. Dropping 10 µl of this solution in parallel with a 10^8^ pfu phage suspension on a bacterial PpG1 lawn confirmed the presence of the EPS degrading activity within the tail spike protein of ϕ15, as the recombinantly purified tail spikes formed identical looking opaque halo zones as those found around the lytic zone caused by phage infection. Further microscopical analysis of these halo zones and the outside bacterial zone clearly shows that the EPS material keeps bacteria closely associated within small cell clusters. In contrast, virtually all bacteria within the halo zone are separated from each other, their EPS material is reduced and sometimes completely removed (Figure 5).

### Role of the viral tail spike in biofilm degradation

To differentiate between the individual contributions of ϕ15 in bacterial cell lysis and EPS depolymerization to biofilm degradation, we attempted to isolate PpG1 strains which are resistant to phage infection, but susceptible for halo formation. However, all of twelve infection resistant strains were also resistant for halo formation. This observation indicates that, although resistance development can occur through various mechanisms (deletion or mutation of receptor sites, host DNases, …), modification or loss of the EPS receptor must be the most straightforward. This again suggest that the EPS receptor is probably a primary and essential receptor for ϕ15 infection, as supported by the specific host range of the phage.

In a last step, phage ϕ15 particles were inactivated by UV radiation mutagenesis, which rendered the particle non-infective but left the enzymatic activity of viral tail spike proteins unharmed. When these particles were dropped onto a bacterial lawn, an opaque halo zone appeared after overnight incubation at 30°C. Further capsule staining of bacteria isolated from these halo zones confirmed the individual arrangement of cells within these zones. Finally, addition of UV inactivated particles (10^2^, 10^4^ and 10^6^ pfu) to 24 h old PpG1 and RD5PR2 biofilms did not influence the pregrown biofilm at the different time points measured after inoculation (2, 4, 8 and 24 h; data not shown).

## Discussion

The aim of this study was to investigate the *in vitro* biofilm degradation capacity of the lytic *P. putida* phage ϕ15, a ‘T7-like virus’ with an EPS depolymerase associated with its tail. Our results suggest that EPS material serves as a primary bacterial receptor for phage adsorption, and that specific adsorption to and disruption of this receptor is necessary to accomplish the phage replication cycle. This was also observed for phages infecting the capsulated *Vibrio cholera* O139 strain [40], *Escherichia coli* K95 [41] and K1 strains [42] and two *Enterobacter agglomerans* strains [43].

A central observation in this study were the characteristic expanding opaque halo zones, which surrounded all ϕ15 plaques. Sixty years ago, these halo zones were already described as an indicator for the presence of phage-associated EPS depolymerases [44]. The observed presence of both phage and viable bacteria in these halo zones can be explained by the need for actively dividing cells for phage replication. When the bacterial population enters the stationary phase of growth, phage replication often ceases or slows down substantially but their tail spikes are still capable of depolymerizing bacterial EPS creating a zone of increased transparency, the halo zone. This zone will increase in diameter over time, as phage diffuse out of the lysis zone of primarily infected bacteria. Based on this observation, one can hypothesize that halo formation is not only caused by the excess of EPS depolymerases produced inside the host during phage replication and released upon bacterial lysis as proposed previously [25], [45]–[47], but also by viral diffusion out of the lysis zone. In that case, differences in halo sizes between different phages are not only dependent on the amount of free enzymatic active spikes [45]–[47], but also on the number of phage produced in one single plaque, which is dependent on the burst size, latent period and adsorption efficiency of that specific phage.

When studying the interaction between phage and biofilm-embedded bacteria, we noticed a time and dose dependent response for ϕ15-mediated biofilm degradation for 24 h and 48 h old biofilms of PpG1 and RD5PR2, with maximal biofilm degradation 8 h after the higher phage doses (10^4^ and 10^6^) were added (only observed for 10^2^ phage when added to the 24 h old biofilm of the highly susceptible PpG1 strain). Similar observations were made in *E. coli* biofilms, exposed to different doses of phage T4, a phage without any EPS depolymerases [48] and to both wild-type phage T7 and its engineered derivative T7_DspB_, which is responsible for an intracellular expression of biofilm-degrading enzymes during its infection [49]. Likewise, a time responsive relative biomass removal was observed for all different aged *Pseudomonas fluorescens* biofilms (up to 168 h) subjected to initial 10^7^ pfu of phage ϕIBB-PF7A [50]. Based on these observations, we can deduce that independent of the presence or absence of EPS depolymerases, time and dose responsive behavior of phage-mediated biofilm disruption seems a common characteristic and this biofilm degradation can probably be increased upon the presence of EPS depolymerase enzymes. Moreover, the maximal biofilm degradation is even dose dependent in the presence of very efficiently replicating phage [*e.g.* the maximal attained phage titer (±7.5 log_10_) for the PpG1 biofilms was reached only 2 h after phage inoculation for all applied titers], with higher initial phage titers ensuring a greater maximal biofilm destruction.

Another important and *in vivo* very variable parameter is the age of the pregrown biofilm. An older biofilm is usually thicker (*e.g.* 48 h old PpG1 biofilms are about 2.5 times thicker than 24 h old biofilms), which makes biofilm degradation more difficult. However, as a biofilm ages, also different phenotypic variants (which can become phage resistant due to loss or change of outer membrane receptors, hereby causing phages to become ‘trapped’ in the biofilm layers) develop within the biofilm layers [51] and due to diffusion constraints of oxygen and nutrients, cells in the lower part of the biofilm become less metabolic active [52], [53]. These two factors may lead to reduced phage susceptibility, depending on the location within the biofilm. Within this study, we noted a marked decrease in phage susceptibility of the PpG1 biofilm with increasing age, while RD5PR2 biofilms retained their susceptibility. Age also influenced the susceptibility of *P. fluorescens* biofilms subjected to phage ϕIBB-PF7A, with the older biofilms (120 and 168 h) being the least susceptible [50]. Similar to our RD5PR2 observations, Hanlon *et al.* [54] did not report a significant decrease in susceptibility of *P. aeruginosa* biofilms to phage F116 with age, as F116 was also very effective against 20-day-old biofilms. In all medical, food and industrial environments, where phage therapy would find its application, it is of great importance that phages are capable of degrading biofilms of different ages. As observed in this work, maintenance of high phage susceptibility over a wide age range of biofilms formed by one host, does not ensure the same results for another host exposed to that same phage. In contrast to biofilm degradation, ϕ15 amplification was not influenced by the age of the preformed *P. putida* biofilms, which was also observed by Sillankorva *et al.* [50]. Probably, this increase in phage numbers is predominantly due to amplification in the planktonic culture which is in equilibrium with the biofilm. The physiological state of these cells is rather similar independent of the age of the biofilm. This also explains that the decrease in planktonic survival occurred rather in parallel with the phage amplification, although the extent at which this occurred was still influenced by the phage-susceptibility of the biofilm.

From these results, it is clear that the extrapolation of phage-planktonic susceptibility for evaluation of phages for therapeutic purposes would be misleading, as observed with the high planktonic, yet low biofilm susceptibility of strain RD5PR2 for phage ϕ15. One can hypothesize that the resistance of the RD5PR2 biofilm for phage infection and subsequent degradation in comparison with the high planktonic susceptibility can be explained by diffusion constraints within the biofilm. ϕ15 forms a haloed plaque with infection of PpG1 and RD5PR2 strains indicating that the viral tail spike can break down the EPS produced by both strains. Hypothetically, a reduced activity of the viral EPS depolymerase on the RD5PR2 EPS material and/or different exopolymer composition with a higher DNA or protein content to EPS could strengthen the RD5PR2 biofilm compared to PpG1, hereby leading to a hampered flow through of phage and consequently a reduced RD5PR2 biofilm degradation. This could be tested for by measuring the diffusion of phage through the biofilm. An additional explanation lies in the phage amplification characteristics (adsorption, latent period and burst size). Phage ϕ15 shows poor adsorption (k_1 min_ = 2.23*10^−9^) on RD5PR2 and an inefficient infection cycle with a occasional release of phage particles starting 90 min post infection but without a defined burst size or latent period, while having a very efficient infection cycle on PpG1. Consistent with this observation, phage amplification on the RD5PR2 biofilms occurred with a constant increase in phage count with prolonged incubation period, reaching its maximum count of about 7 log_10_ 24 h after phage inoculation, whereas on PpG1 a maximum was rapidly reached. This suggests that the efficiency of the infection cycle on a specific host forms an important criterion for phage selection. Carson *et al.* [55] came to similar conclusions when using the same coli-proteus phage in biofilm experiments: the 24 h old *E. coli* ATCC 11303 biofilm was reduced by three-log titers, while a *Proteus mirabilis* biofilm diminished by only an approximate one-log.

One of the main concerns with regard to phage therapy is the development of phage resistance. Contrary to some papers which reported increasing phage-mediated biofilm destruction for incubation periods longer 24 h [49], [55], we clearly observed an increase in biofilm and planktonic survival after 24 h for every initial phage titer added. This was probably caused by bacterial resistance development against phage infection as was previously observed in other phage-biofilm studies [56]. Because of the high bacterial mutation rate, bacteria can become resistant for phage infection within a short period of time. In addition phage have a lower coevolutionary potential than their bacterial hosts [57], giving the bacterial resistant mutants the opportunity to grow thick biofilms. Lacqua *et al.* [56] even found that after exposure of an *E. coli* biofilm to two lytic phages (OP7061 and OP10081), each emerging phage-resistant strain showed an increased clumping in liquid media, biofilm formation and production of fimbria-like extracellular structures compared to the wild type. This suggests that growth as a biofilm might even be one of the mechanisms for phage resistance. In addition, although persister cells (cells characterized by a transient phase of slow growth or dormancy) do not survive infections by lytic phages, they are only killed when the persisters switch to normal growth and the lytic process is able to proceed [58]. This phenomenon can delay the bacterial cell lysis from minutes to hours and can hereby delay the phage-mediated degradation of the biofilm community.

Phage-mediated biofilm degradation is brought about by bacterial lysis on the one hand and EPS depolymerization on the other hand, which probably act synergistically. The absence of biofilm degradation caused by viral attached EPS depolymerases alone, as observed here upon usage of UV inactivated phage particles, does not rule out the essential contribution of these EPS depolymerases in phage-mediated biofilm degradation. One can hypothesize that phage need their viral EPS depolymerization activity to tunnel through the biofilm to gain access to neighboring host cells, without completely disrupting the biofilm as this would also remove their potential new hosts. Complete absence of EPS depolymerization activity would therefore reduce biofilm degradation activity by phage as phage diffusion through the biofilm would be hampered. This is demonstrated for *P. aeruginosa* phage E79, which doesn't have any EPS depolymerases and was more successful at infecting surface cells than cells at depth in the biofilm [59].

It is generally recognized that bacteria predominantly live in biofilms. With regard to phage therapy, phages with viral-attached EPS depolymerases should therefore preferably be selected for, as they are after all able to break down biofilms by attacking two of their main constituting parts, bacterial cells and EPS material. However, one of the major hurdles with regard to phage therapy still to overcome is their narrow host range, conferred by the high specificity of their associated EPS depolymerases [43], [60], [61]. This restricts the phage in infecting only a very few strains of one single species. As observed in our study, phage ϕ15 only infects seven *P. putida* strains out of a selection of 53 but conferred also a marked difference in breaking down PpG1 and RD5PR2 biofilms. Therefore, one should first carefully identify the bacterial pathogens before therapy with a highly lytic phage with a proper viral attached EPS depolymerase can be started. The chance of finding such a specific phage is however likely to be low, and nonetheless bacteria easily develop resistance to phages. From this point of view, application of ‘phage cocktails’ directed at numerous strains of the target species in combination with the concept of ‘engineering’ highly lytic phages with the appropriate EPS degrading enzymes are powerful tools opening new perspectives in phage therapy.

## Supporting Information

Figure S1 **Relative biofilm forming capacities of a collection of 53** ***P. putida*** **strains.** The average biofilm formation of each strain (A_x_) is the result of eight independent experiments with A_0_ being the uninoculated control sample. For each strain, the average relative biofilm forming capacity ((A_x_-A_0_)/Average(A_n_-A_0_)) and standard deviation is given.
(TIF)

Figure S2 **Electron microscopic image of negatively stained** ***P. putida*** **phage ϕ15 particles.**
(TIF)

Figure S3 **Microbiological characteristics of phage ϕ15.** (A) pH-stability. Phage counts (P) are validated after a 24 h exposure at room temperature and given relative to a control sample (P_0_). For each pH, the average and standard deviation of three independent experiments is given. (B) Adsorption curves of ϕ15 on its host PpG1 (light gray) and on the RD5PR2 strain (dark gray). Values are given relative to phage number at time point zero. In each case, the average and standard deviation of three independent experiments is given. (C+D) One-step growth curves of ϕ15 on PpG1 (C) and on RD5PR2 (D) showing the amount of phage (P) released relative to the initial phage number (P_0_) at time point zero. In each case, the average and standard deviation of three independent experiments is given.
(TIF)

Figure S4 **Genomic map of ϕ15 and gh-1.** Genes, in the three forward (left to right) reading frames, are represented by filled grey boxes depending on the percentage of amino-acid identity between ϕ15 and gh-1, while white boxes indicate unique genes to one phage. The same gene numbering system, starting from left to right in the genomic sequence, as that of T7 was used. Genes which only have sequence similarity to the ‘T7-like virus’ gh-1 are simply named ‘gh-1/’ followed with the similar gene number. Genes that are not present or have no sequence similarity to a previously characterized T7-like phage are named ‘ϕ15/’ with gene numbering also from left to right in the genome. Experimentally confirmed structural proteins are marked with an asterisk. The host and phage specific RNA polymerase promoters are indicated by double and single arrowheads, respectively, indicating the orientation of transcription. White spheres above the map indicate ρ-independent terminators.
(TIF)

Figure S5 **CLUSTALW alignment of the phage RNAP of ϕ15, gh-1 and T7.** Amino-acid sequences of phage RNAP that are responsible for specific recognition and binding to the phage promoter sequence (ϕ15: 737–769) and for making additional contacts with the promoter sequence (ϕ15: 90–101) were aligned.
(TIF)

Table S1 **Putative genes of ϕ15 and their homologies to gh-1.** The same gene numbering system, starting from left to right in the genomic sequence, as that of T7 was used. Genes which only have sequence similarity to gh-1 are simply named ‘*gh-1/*’ followed with the similar gene number. Genes that are not present or have no sequence similarity to a previously characterized T7-like phage are named ‘*ϕ15/*’ with gene numbering from left to right in the genome. 44 of the 50 potential genes contain a AUG initiation codon, while GUG is used for the remaining six ORFs. All three stop codons are used in ϕ15, with UAA being the most frequent. The use of the second codon GCU (alanine) in high expressed proteins (Gp2.5, Gp3, Gp3.5, Gp8, Gp9, Gp10, Gp12, Gp15 and Gp17) as found within T7, ϕYeO3-12 and ϕSG-JL2, is not completely conserved within ϕ15. Gp8 and Gp10 with GCA and Gp15 with GCC, also encode for alanine, while Gp9 and Gp12 have serine and proline as second amino-acid, respectively.
(DOC)

Table S2 **Structural proteome of phage ϕ15.**
(DOC)

Table S3 **Predicted promoter sequences in the genome of ϕ15.** The *P. putida* host promoter sequences are given and their −10 and −35 boxes are underlined as well as the transcription start at +1. In the second part of the table, the ϕ15 promoter sequences are aligned and a consensus sequence is presented. Also an alignment of consensus promoter sequences of all classified ‘T7-like viruses’ in the NCBI database is presented. Conserved nucleotides, when compared with the ϕ15 consensus are marked in grey.
(DOC)

Table S4 **Predicted σ-independent terminators in the genome of ϕ15.** Paired nucleotides in the stem-loop structure are underlined.
(DOC)

Text S1 **Genome organization and analysis of phage ϕ15.**
(DOC)
