# Supplementary figures and images for: The T7-Related Pseudomonas putida Phage ϕ15 Displays Virion-Associated Biofilm Degradation Properties
Source: PLoS One. 2011 Apr 19;6(4):e18597. doi: 10.1371/journal.pone.0018597 (PMC3079711; doi:10.1371/journal.pone.0018597)

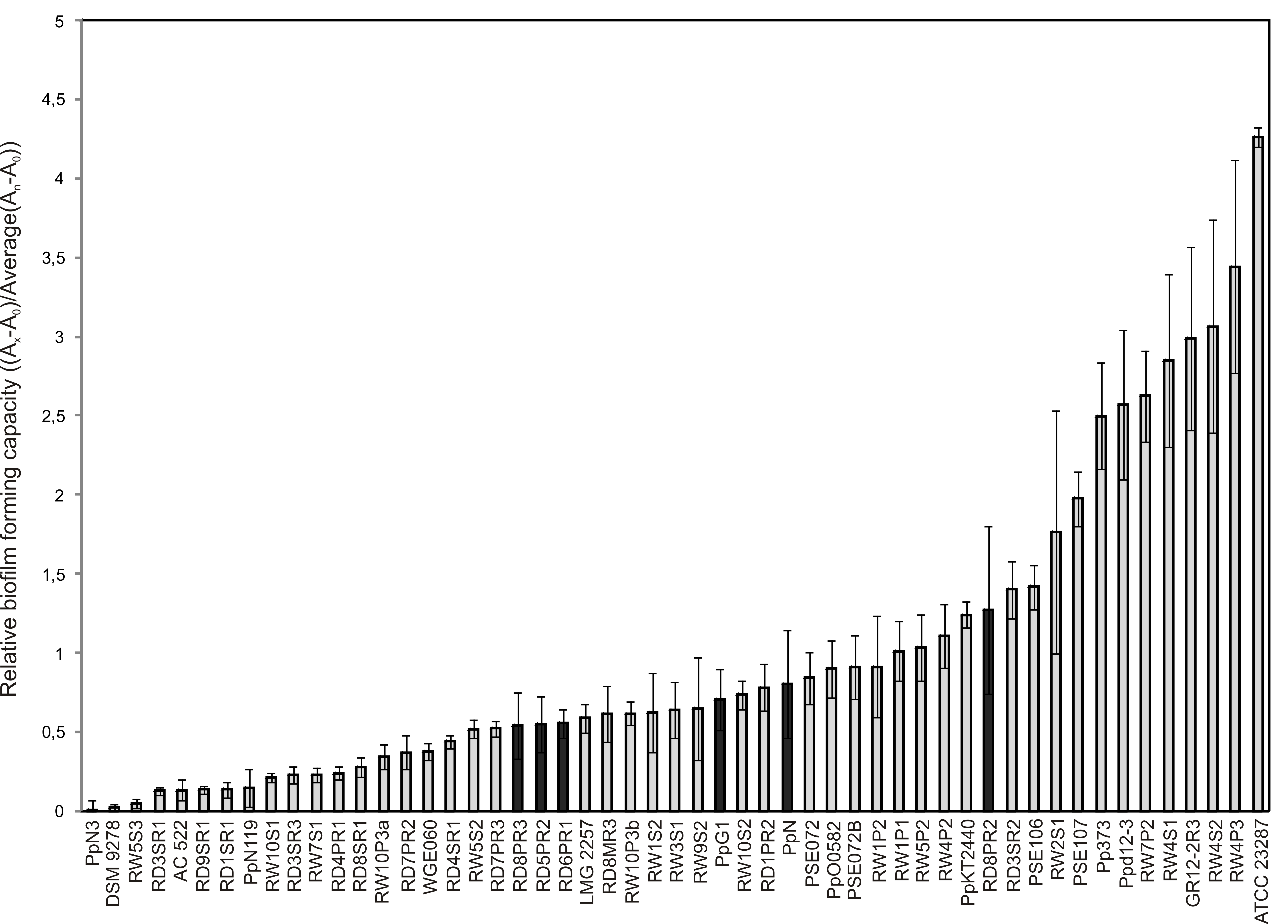

Supplement: Figure S1 — Relative biofilm forming capacities of a collection of 53 P. putida strains. The average biofilm formation of each strain (Ax) is the result of eight independent experiments with A0 being the uninoculated control sample. For each strain, the average relative biofilm forming capacity ((Ax-A0)/Average(An-A0)) and standard deviation is given. (TIF) [file pone.0018597.s001.tif]

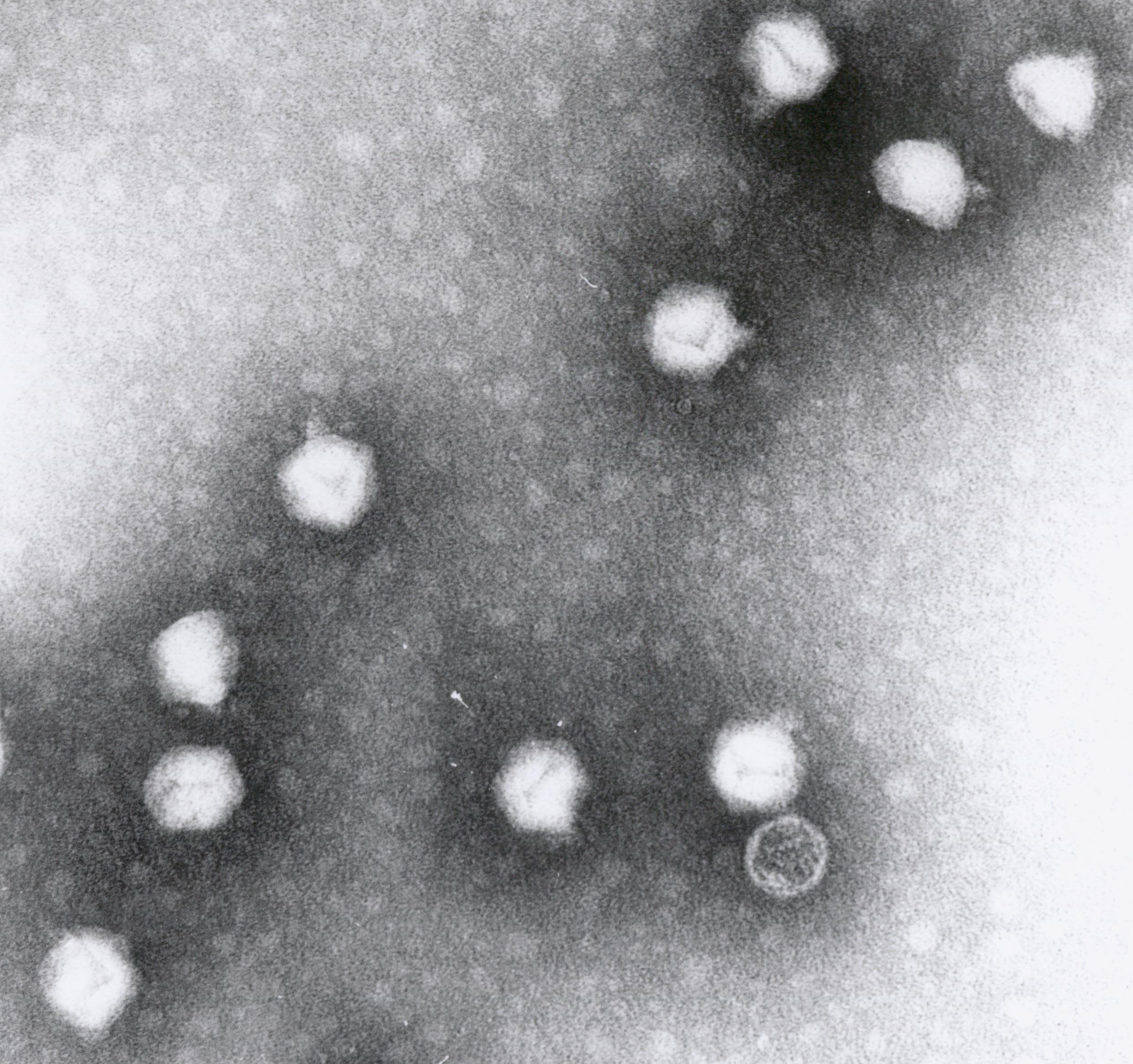

Supplement: Figure S2 — Electron microscopic image of negatively stained P. putida phage ϕ15 particles. (TIF) [file pone.0018597.s002.tif]

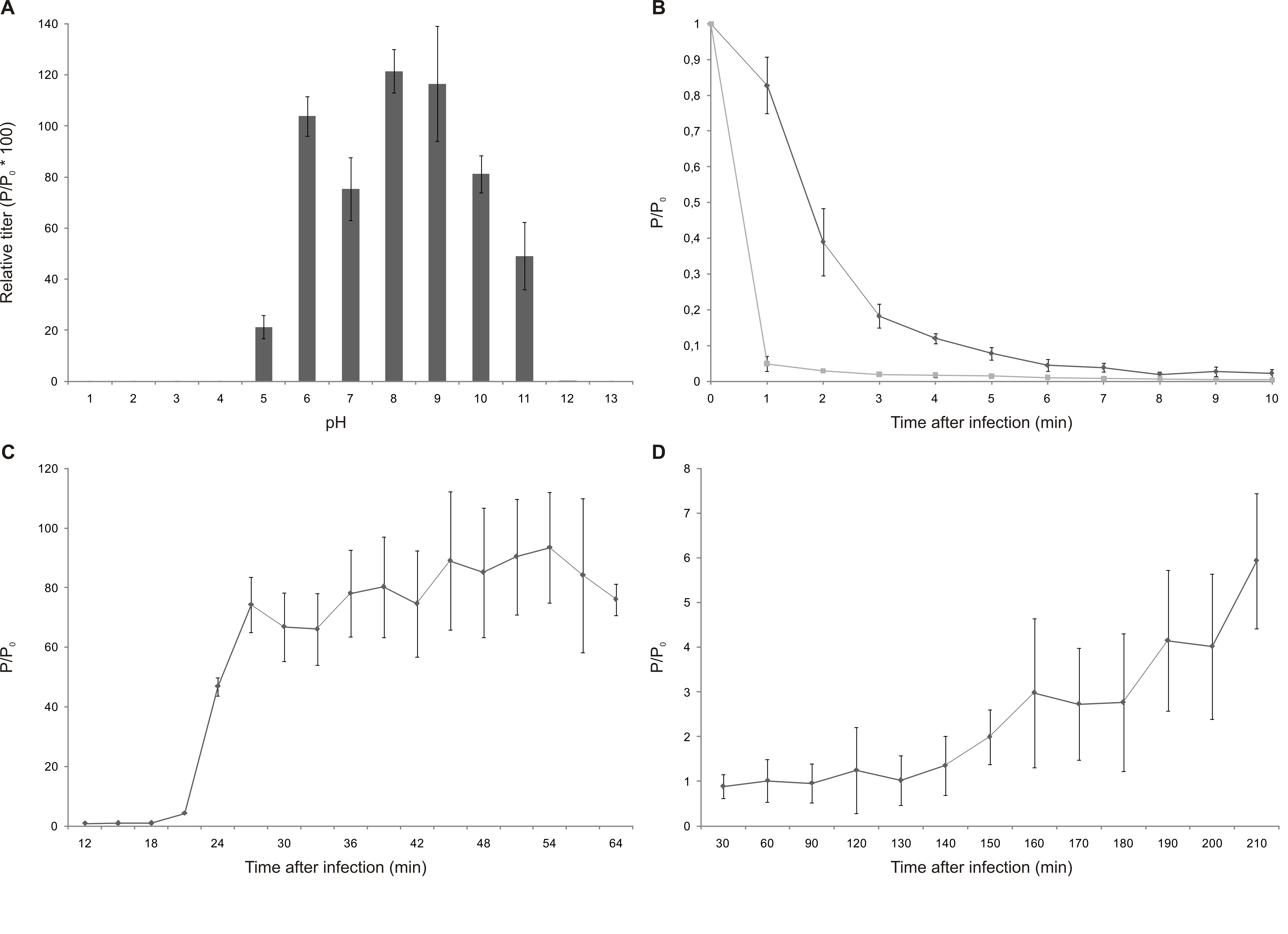

Supplement: Figure S3 — Microbiological characteristics of phage ϕ15. (A) pH-stability. Phage counts (P) are validated after a 24 h exposure at room temperature and given relative to a control sample (P0). For each pH, the average and standard deviation of three independent experiments is given. (B) Adsorption curves of ϕ15 on its host PpG1 (light gray) and on the RD5PR2 strain (dark gray). Values are given relative to phage number at time point zero. In each case, the average and standard deviation of three independent experiments is given. (C+D) One-step growth curves of ϕ15 on PpG1 (C) and on RD5PR2 (D) showing the amount of phage (P) released relative to the initial phage number (P0) at time point zero. In each case, the average and standard deviation of three independent experiments is given. (TIF) [file pone.0018597.s003.tif]

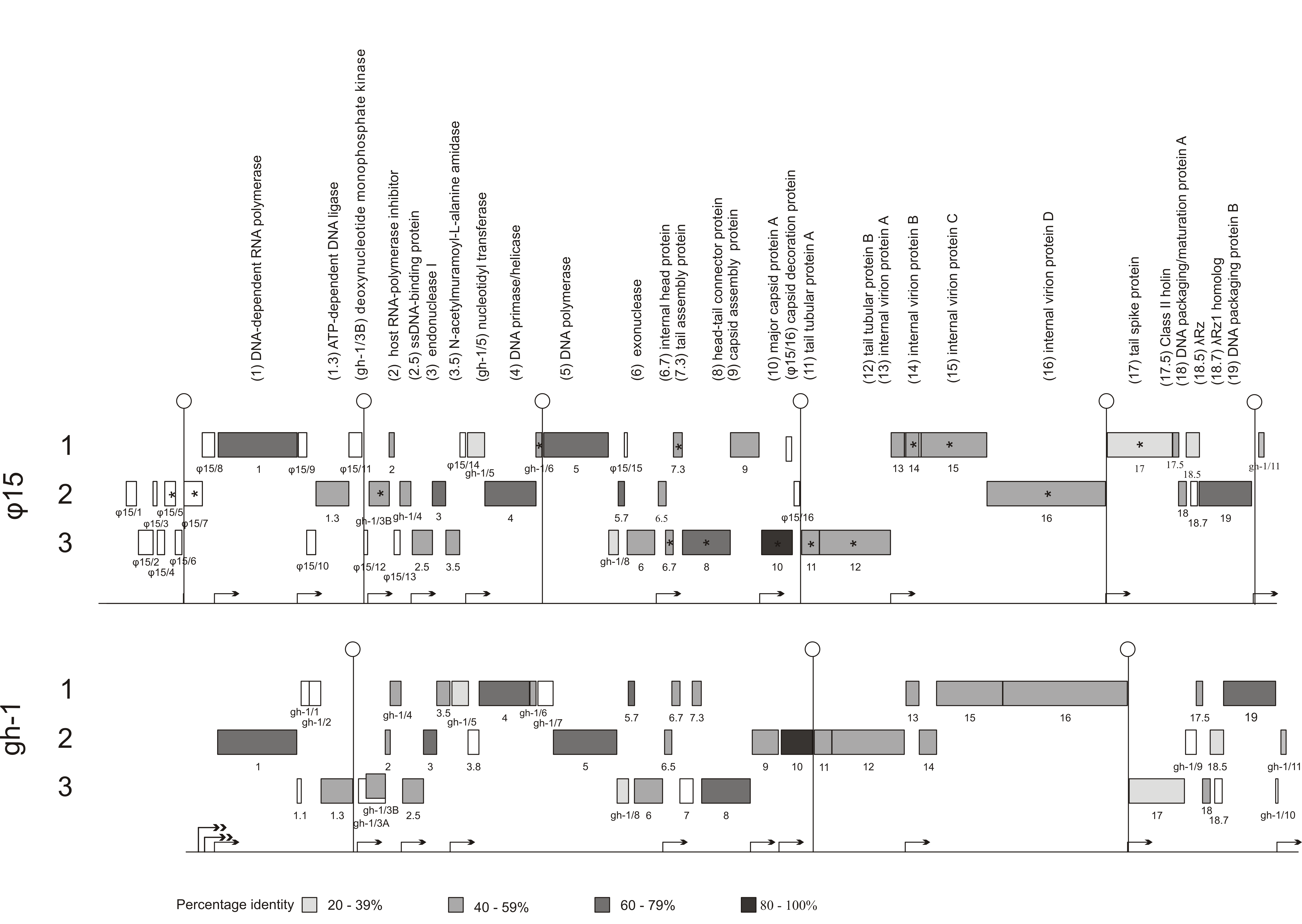

Supplement: Figure S4 — Genomic map of ϕ15 and gh-1. Genes, in the three forward (left to right) reading frames, are represented by filled grey boxes depending on the percentage of amino-acid identity between ϕ15 and gh-1, while white boxes indicate unique genes to one phage. The same gene numbering system, starting from left to right in the genomic sequence, as that of T7 was used. Genes which only have sequence similarity to the ‘T7-like virus’ gh-1 are simply named ‘gh-1/’ followed with the similar gene number. Genes that are not present or have no sequence similarity to a previously characterized T7-like phage are named ‘ϕ15/’ with gene numbering also from left to right in the genome. Experimentally confirmed structural proteins are marked with an asterisk. The host and phage specific RNA polymerase promoters are indicated by double and single arrowheads, respectively, indicating the orientation of transcription. White spheres above the map indicate ρ-independent terminators. (TIF) [file pone.0018597.s004.tif]

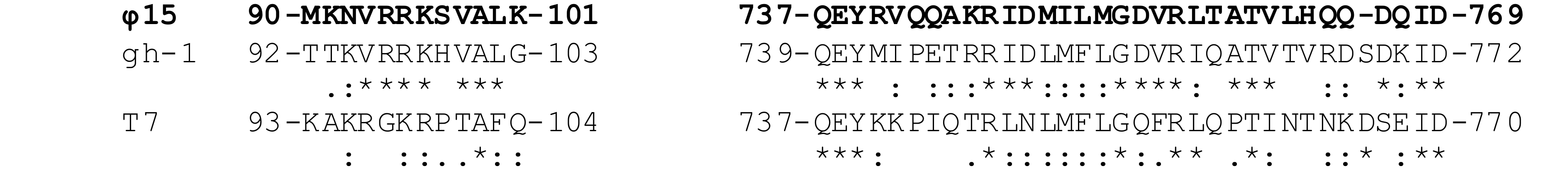

Supplement: Figure S5 — CLUSTALW alignment of the phage RNAP of ϕ15, gh-1 and T7. Amino-acid sequences of phage RNAP that are responsible for specific recognition and binding to the phage promoter sequence (ϕ15: 737–769) and for making additional contacts with the promoter sequence (ϕ15: 90–101) were aligned. (TIF) [file pone.0018597.s005.tif]
