# Supplementary material for: The T7-Related Pseudomonas putida Phage ϕ15 Displays Virion-Associated Biofilm Degradation Properties
Source: PLoS One. 2011 Apr 19;6(4):e18597. doi: 10.1371/journal.pone.0018597 (PMC3079711; doi:10.1371/journal.pone.0018597)
Supplement: Table S1 — Putative genes of ϕ15 and their homologies to gh-1. The same gene numbering system, starting from left to right in the genomic sequence, as that of T7 was used. Genes which only have sequence similarity to gh-1 are simply named ‘gh-1/’ followed with the similar gene number. Genes that are not present or have no sequence similarity to a previously characterized T7-like phage are named ‘ϕ15/’ with gene numbering from left to right in the genome. 44 of the 50 potential genes contain a AUG initiation codon, while GUG is used for the remaining six ORFs. All three stop codons are used in ϕ15, with UAA being the most frequent. The use of the second codon GCU (alanine) in high expressed proteins (Gp2.5, Gp3, Gp3.5, Gp8, Gp9, Gp10, Gp12, Gp15 and Gp17) as found within T7, ϕYeO3-12 and ϕSG-JL2, is not completely conserved within ϕ15. Gp8 and Gp10 with GCA and Gp15 with GCC, also encode for alanine, while Gp9 and Gp12 have serine and proline as second amino-acid, respectively. (DOC) [file pone.0018597.s006.doc]

| **Gene**  **Table S1. Putative genes of φ15 and their homologies to gh-1.** | **Start-Stop^a^** | **Shine-Dalgarno^b^** | **Stopcodon** | **Size (kDa)/pI/GC-%** | | **Function** | **% AA^c^ identity^d^** | | **Comments^z^** |
| --- | --- | --- | --- | --- | --- | --- | --- | --- | --- |
| ***φ15/1*** | 947-1261 | **gaggag**cgacaccuaaug | tga | 12.5/9.22/55.9 |  | |  |  | |
| ***φ15/2*** | 1329-1820 | **gaga**uagcuaccaug | taa | 18.4/5.09/57.9 |  | |  | homology with LKA1/Gp7 2.0E-18 | |
| ***φ15/3*** | 1820-1951 | **gaaggg**cgccuguaaug | tga | 5.1/8.46/53.8 |  | |  | 1 TM | |
| ***φ 15/4*** | 1962-2207 | **aaagg**ugacccaaug | taa | 9.3/6.83/59.8 |  | |  |  | |
| ***φ 15/5*** | 2213-2572 | **aaggag**uaacaaccaug | taa | 13.4/8.86/59.4 |  | |  |  | |
| ***φ 15/6*** | 2565-2789 | **ggagg**cccgugaaaug | taa | 8.2/9.74/58.7 |  | |  |  | |
| ***φ 15/7*** | 2858-3475 | **gaaggg**cauccucaug | tga | 25.0/7.17/58.4 |  | |  |  | |
| ***φ 15/8*** | 3472-3888 | **ggaggaaa**ugguaug | tag | 15.9/6.07/60.0 |  | |  |  | |
| ***1*** | 4009-6657 | **gagg**cccagcaug | taa | 99.8/6.64/58.0 | DNA-dependent RNA polymerase | | 61.8 |  | |
| ***φ 15/9*** | 6700-6984 | **aggagaaa**cacaaug | tga | 10.7/10.0/60.4 |  | |  | low homology with T7/Gp1.1 8.4 | |
| ***φ 15/10*** | 6981-7283 | **aaaaggag**cgugcacugug | taa | 11.5/6.09/56.4 |  | |  |  | |
| ***1.3*** | 7295-8395 | **aaaggaga**ccagaaug | taa | 40.8/5.94/60.1 | ATP-dependent DNA ligase | | 40.3 |  | |
| ***φ 15/11*** | 8401-8835 | **aaggag**uaaccauugug | taa | 15.6/9.21/58.4 |  | |  | 1 TM; SP 21-22 | |
| ***φ 15/12*** | 8901-9026 | **aaggaga**cuugacaug | taa | 4.5/4.36/56.3 |  | |  | 1 TM | |
| ***gh-1/3B*** | 9080-9754 | **agaggaga**uuauuuccgug | tga | 25.5/5.47/59.3 | deoxynucleotide monophosphate kinase | | 47.6 |  | |
| ***2*** | 9751-9921 | **aagggg**uagacguaug | tga | 6.5/4.95/55.6 | host RNA polymerase inhibitor | | 42.9 |  | |
| ***φ 15/13*** | 9918-10118 | **aaggagg**ucgaaug | tga | 7.5/4.33/55.7 |  | |  |  | |
| ***gh-1/4*** | 10115-10480 | **ggagg**uggccaug | taa | 13.5/7.95/59.6 |  | | 42.1 |  | |
| ***2.5*** | 10530-11204 | **aaggagaaa**cacaaug | tga | 25.0/4.71/58.8 | ssDNA-binding protein | | 48.7 | strongest homology with K11/Gp2.5 3.0E-55 | |
| ***3*** | 11204-11647 | **gacgacgac**uucugaug | taa | 16.6/9.41/55.6 | endonuclease I | | 60.5 |  | |
| ***3.5*** | 11658-12119 | **aaggaggg**ccguaug | tag | 16.8/7.78/56.7 | N-acetylmuramoyl-L-alanine amidase | | 58.8 |  | |
| ***φ 15/14*** | 12130-12315 | **agaggagga**cguaug | taa | 7.0/9.03/56.5 |  | |  |  | |
| ***gh-1/5*** | 12385-12957 | **agaggaga**cuauuuccgaug | taa | 21.3/4.85/57.9 | nucleotidyl transferase | | 34.0 |  | |
| ***4*** | 12968-14683 | **aaggag**ucccuaaug | tga | 63.4/5.32/58.0 | DNA primase/helicase | | 66.3 |  | |
| ***gh-1/6*** | 14683-14880 | **gaaagaaggaga**uuucugaug | tga | 7.4/10.35/59.1 |  | | 53.7 | CC | |
| ***5*** | 14944-17112 | **agaaggagga**cgacaug | taa | 80.6/7.35/58.2 | DNA polymerase | | 63.3 | CC | |
| ***gh-1/8*** | 17130-17450 | **agaggaga**ccuaucaaaug | tga | 11.6/6.6/61.4 |  | | 25.0 |  | |
| ***5.7*** | 17447-17656 | **gaagg**ugcgcgaaug | tga | 7.8/9.52/56.7 |  | | 76.8 |  | |
| ***φ 15/15*** | 17653-17751 | **ggagg**ugcacugug | tga | 3.9/8.37/53.5 |  | |  |  | |
| ***6*** | 17748-18671 | **aaggagg**ugacccagug | taa | 35.1/5.11/58.4 | Exonuclease | | 55.9 |  | |
| ***6.5*** | 18794-19054 | **aagaggagg**cauucaug | taa | 9.5/4.43/54.4 |  | | 48.8 |  | |
| ***6.7*** | 19038-19292 | **ggagga**ccuaccgaaug | taa | 8.8/8.85/58.8 | internal head protein | | 43.0 |  | |
| ***7.3*** | 19303-19596 | **aaggaga**ccauuaug | taa | 9.7/10.12/59.9 | tail assembly protein | | 53.4 | strongest homology with K11/Gp7.3 6.0E-9 | |
| ***8*** | 19608-21209 | **aaggagg**ugagcuaug | taa | 58.4/4.68/59.1 | head-tail connector protein | | 72.6 |  | |
| ***9*** | 21220-22176 | **aaggaga**cauaaaug | taa | 34.4/4.32/60.1 | capsid assembly protein | | 47.5 |  | |
| ***10*** | 22266-23294 | **aaggagaa**cuacauaug | taa | 36.0/6.39/60.3 | major capsid protein | | 80.1 |  | |
| ***φ 15/16*** | 23354-23542 | **gaaga**cgaug | tga | 6.2/4.09/58.7 | capsid decoration protein | |  |  | |
| ***11*** | 23610-24200 | **aggaggag**cuaug | taa | 22.0/4.45/57.0 | tail tubular protein A | | 58.7 |  | |
| ***12*** | 24210-26591 | **aaggaggg**cauaug | tga | 87.8/5.81/57.4 | tail tubular protein B | | 59.5 |  | |
| ***13*** | 26617-27057 | **agggagac**cacuaug | taa | 17.0/9.56/60.5 | internal virion protein A | | 41.1 |  | |
| ***14*** | 27067-27615 | **aggagg**uucuaug | taa | 19.7/8.73/58.5 | internal virion protein B | | 47.7 |  | |
| ***15*** | 27625-29826 | **aagagg**cacaaug | taa | 81.2/5.3/59.8 | internal virion protein C | | 41.6 | CC | |
| ***16*** | 29837-33817 | **aaggagaa**cccuaug | taa | 142.5/6.03/60.6 | internal virion protein D | | 50.1 | transglycosylase SLT domain 4.0E-9 | |
| ***17*** | 33877-36060 | **aaggagaa**cacaaug | taa | 77.8/6.23/58.1 | tail spike protein | | 25.0 | strongest homology with T7/Gp17 4.0E-35  Similarity restricted to the first 147 AA | |
| ***17.5*** | 36076-36288 | **aaaggagga**cacaug | taa | 7.5/6.15/54.9 | class II holin | | 40.3 | strongest homology with φSG-JL2/Gp17.5 3.0E-5; 2 TM | |
| ***18*** | 36272-36532 | **aaggagaa**ccgcaaug | tga | 9.8/4.7/57.1 | DNA packaging/maturation protein A | | 47.1 |  | |
| ***18.5*** | 36529-36972 | **gaaggagaaa**ccauccaaug | taa | 16.5/5.97/57.4 | λR_z_ homolog, endopeptidase | | 31.2 | strongest homology with Yepe2/Gp18.5  3.0E-8; 1TM | |
| ***18.7*** | 36680-36904 | **gagaaggag**cgugacgaug | taa | 8.2/10.35/55.1 | λR_zI_ homolog | |  | strongest homology with Yepe2/Gp18.7  5.0E-5; 1TM; SP 37-38 | |
| ***19*** | 36962-38722 | **aggagg**ugacccgug | tag | 65.9/5.36/58.9 | DNA packaging/maturation protein B | | 72.3 | 1 TM; CC | |
| ***gh-1/11*** | 38965-39141 | aagggagaccaug | tag | 5.9/9.59/55.4 |  | | 43.3 | 1 TM; SP 19-20; CC | |

^a^ Nucleotide positions corresponding to the first nucleotide of the initiation codon and the last nucleotide of the stop codon.

^b^ Shine-Dalgarno sequences are marked in bold, initiation codons are underlined

^c^ AA, amino-acids

^d^ Pairwise amino –acid identities of homologues proteins of gh-1 and φ15 were conducted with the GeneStream Align algorithm using default parameters [14].

^e^ Protein homology and protein domains were determined using BlastP [3] and Pfam [7]. TM, transmembrame helices, SP, signal peptide and CC, coiled coils were predicted using TMHMM [8], SignalP [10] and the

COILS algorithm [9].

The same gene numbering system, starting from left to right in the genomic sequence, as that of T7 was used. Genes which only have sequence similarity to gh-1 are simply named ‘*gh-1/*’ followed with the similar gene number. Genes that are not present or have no sequence similarity to a previously characterized T7-like phage are named ‘*φ15/*’ with gene numbering from left to right in the genome. 44 of the 50 potential genes contain a AUG initiation codon, while GUG is used for the remaining six ORFs. All three stop codons are used in φ15, with UAA being the most frequent. The use of the second codon GCU (alanine) in high expressed proteins (Gp2.5, Gp3, Gp3.5, Gp8, Gp9, Gp10, Gp12, Gp15 and Gp17) as found within T7, φYeO3-12 and φSG-JL2, is not completely conserved within φ15. Gp8 and Gp10 with GCA and Gp15 with GCC, also encode for alanine, while Gp9 and Gp12 have serine and proline as second amino-acid, respectively.
