# Supplementary material for: The T7-Related Pseudomonas putida Phage ϕ15 Displays Virion-Associated Biofilm Degradation Properties
Source: PLoS One. 2011 Apr 19;6(4):e18597. doi: 10.1371/journal.pone.0018597 (PMC3079711; doi:10.1371/journal.pone.0018597)
Supplement: Table S2 — Structural proteome of phage ϕ15. (DOC) [file pone.0018597.s007.doc]

| **Gene** | **Putative function** | **No. of peptides** | **Sequence identity (%)** |
| --- | --- | --- | --- |
| ***φ 15/5*** | unknown | 2 | 16.1 |
| ***φ 15/7*** | unknown | 1 | 5.5 |
| ***gh-1/3B*** | deoxynucleotide monophosphate kinase | 2 | 11.2 |
| ***gh-1/6*** | unknown | 2 | 39.1 |
| ***6.7*** | internal head protein | 3 | 68.7 |
| ***7.3*** | tail assembly protein | 3 | 57.3 |
| ***8*** | head-tail connector protein | 24 | 78.8 |
| ***10*** | major capsid protein | 14 | 61.8 |
| ***11*** | tail tubular protein A | 5 | 21.5 |
| ***12*** | tail tubular protein B | 29 | 52.8 |
| ***14*** | internal virion protein B | 6 | 58.0 |
| ***15*** | internal virion protein C | 30 | 58.6 |
| ***16*** | internal virion protein D | 52 | 57.9 |
| ***17*** | tail spike protein | 26 | 59.1 |

**Table S2. Structural proteome of phage φ15.**
