# Supplementary material for: The T7-Related Pseudomonas putida Phage ϕ15 Displays Virion-Associated Biofilm Degradation Properties
Source: PLoS One. 2011 Apr 19;6(4):e18597. doi: 10.1371/journal.pone.0018597 (PMC3079711; doi:10.1371/journal.pone.0018597)
Supplement: Table S3 — Predicted promoter sequences in the genome of ϕ15. The P. putida host promoter sequences are given and their −10 and −35 boxes are underlined as well as the transcription start at +1. In the second part of the table, the ϕ15 promoter sequences are aligned and a consensus sequence is presented. Also an alignment of consensus promoter sequences of all classified ‘T7-like viruses’ in the NCBI database is presented. Conserved nucleotides, when compared with the ϕ15 consensus are marked in grey. (DOC) [file pone.0018597.s008.doc]

**Table S3. Predicted promoter sequences in the genome of φ15.**

| **Promoters for** | **Start-End** | **Prior to gene** | **Putative promoter sequence** |
| --- | --- | --- | --- |
| ***P. putida* RNAP**  **A1**  **A2**  **Phage specific RNAP**  **φOL**  **φ(φ15/7)**  **φ1**  **φ(φ15/9)**  **φ(gh-1/3B)**  **φ2.5**  **φ(gh-1/5)**  **φ6.5**  **φ10**  **φ13**  **φ17**  **φOR** | 384-429  675-720  359-381  2829-2851  3871-3839  6654-6676  9024-9046  10478-10500  12312-12334  18714-18736  22188-22210  26589-26611  33815-33837  38770-38792  Consensus | *φ15/1*  *φ15/1*  *φ15/1*  *φ15/7*  *1*  *φ15/9*  *gh-1/3B*  *2.5*  *gh-1/5*  *6.50*  *10*  *13*  *17*  *gh-1/11*    φ**15**  **gh-1**  **VP4**  **T3***  **T7**  φ**A1122**  **K1F**  **Kvp1** | -35 -10 +1  AAGGGGTTGACAGGGGCCTGATTGGTGCGTAAAGTTCACTCCATCGAGAC^a^  ACAACGCTTGACAGGGTCGCCAAATGCTGTAACATGGCCACCAAGCAACA^a^  -14 -7 -4 +1  TAAAAACCCACACAATAGGATCA  CAAATACCCACACTATCGGGAAT  CAAAAACCCACACAATAGGGCGA  TTAAAACCCACACAATAGAAAGA  TAAAAACCCACACAATAGAAAGA  TAAAAACCCACACAATAGAAAGA  CTAAAACCCACACAATAGATGGA  TAAAACCCCACACAATAGACAGA  TAAAAACCCACACAATAGACAGA  TGAAACCACACACAATAGGGAGA  TAAAACCCCACACAATAGATAGG  TAAAAACCCACACAATAGACAGA  -14 -7 -4 +1  YWAAAMCCCACACAATAGRXARA  TAAAAACCCTCACTRTGGCHSCM^b^  XBAAYYAACCCTGACTATAGGRM^c^  AATTAACCCTCACTAAAGGGAGA^d^  TAATACGACTCACTATAGGGAGA^d^  YAATWCGACTCASTAWAGRRRRH^c^  MCTAAACTATCACTATAGGRAAA^e^  YWATWVGACTCACTAWKGGRRRR^c^ |
|  |  |  |  |

^*^ φYeO3-12 [33] and φSG-JL2 [34] have the same phage consensus sequence as phage T3.

^b, d, e^ Consensus sequences retrieved from Kovalyova and Kropinski [23], Imburgo *et al*. [45] and Scholl and Merril [48], respectively.

^c^ Consensus sequence determined based on promoters retrieved from NCBI database.

The *P. putida* host promoter sequences are given and their -10 and -35 boxes are underlined as well as the transcription start at +1. In the second part of the table, the φ15 promoter sequences are aligned and a consensus sequence is presented. Also an alignment of consensus promoter sequences of all classified ‘T7-like viruses’ in the NCBI database is presented. Conserved nucleotides, when compared with the φ15 consensus are marked in grey.
