# Supplementary material for: The T7-Related Pseudomonas putida Phage ϕ15 Displays Virion-Associated Biofilm Degradation Properties
Source: PLoS One. 2011 Apr 19;6(4):e18597. doi: 10.1371/journal.pone.0018597 (PMC3079711; doi:10.1371/journal.pone.0018597)
Supplement: Table S4 — Predicted σ-independent terminators in the genome of ϕ15. Paired nucleotides in the stem-loop structure are underlined. (DOC) [file pone.0018597.s009.doc]

**Table S4. Predicted σ-independent terminators in the genome of φ**15.

| **Terminators** | **Start-End** | **Prior to gene** | **ΔG (kcal/mol)** | | **Stem-loop structure** |
| --- | --- | --- | --- | --- | --- |
| **T_early1_**  **T_early2_/T_E_**  **T_middle_**  **T_late1_/T_Ф_** | 2781-2822  8844-8880  14888-14911  23552-23594 | *φ15/7*  *φ15/12*  *5*  *11* | -11.40  -21.50  -10.5  -23.50 | GGCAUCCUCAUGGGUGCC**UUUU**  GCCUCAUCGACUUCGGUCGGUGGGGC**UUUUUGCGUUU**  GCCUUCCCUUAGUGGAGGGC**UUUU**  CCCCUUGGGUCCCAUAACGGGGCUUGAGGGG**UUUUU** | |
| **T_late2_**  **T_late3_** | 33835-33861  38702-38740 | *17*  *gh-1/11* | -8.50  -17.6 | AGGGCGCUUACAUAGGCCCUCCA**UUUU**  GGGUCCUUUAUCGACUGGUAGAGGGCCCGUAGGG**UUUUU** | |

Paired nucleotides in the stem-loop structure are underlined.
