# Supplementary material for: The T7-Related Pseudomonas putida Phage ϕ15 Displays Virion-Associated Biofilm Degradation Properties
Source: PLoS One. 2011 Apr 19;6(4):e18597. doi: 10.1371/journal.pone.0018597 (PMC3079711; doi:10.1371/journal.pone.0018597)
Supplement: Text S1 — Genome organization and analysis of phage ϕ15. (DOC) [file pone.0018597.s010.doc]

**Materials and Methods**

**Electron microscopic imaging.** Purified bacteriophage samples were deposited on carbon-coated copper grids and negatively stained with 2% (w/v) potassium phosphotungstate (pH 7.0) for visualization by a Philips EM 300 transmission electron microscope.

**Phage DNA isolation and sequencing.** Phage DNA was isolated as described by Naryshkina *et al*. [1]. Initial sequence data were obtained from a shotgun library of phage DNA in pUC18 (ABI 3130 Sequencer, BigDye chemistry, Applied Biosystems). Several rounds of primer walking directly on phage DNA were performed until one single contig with a average fourfold redundancy was generated. Open reading frames (ORF) were identified using ORF Finder (<http://www.ncbi.nlm.nih.gov/projects/gorf>), and the GeneMark.hmm algorithm [2]. Annotated ORF’s were manually inspected for the presence of a convincing Shine-Dalgarno sequence. Translated ORFs were compared with known proteins using BlastP [3] against the non-redundant database considering a E-value threshold of 0.001. In addition, the HHpred server [4,5] was used for protein remote homolog detection and 3D structure prediction in combination with the Phyre structure prediction algorithm [6]. Conserved protein domains were identified using Pfam [7], while prediction of transmembrane helices, coiled coils and signal peptides were performed using the TMHMM [8], the COILS [9] and the SignalP algorithm [10], respectively. Putative tRNA genes and -1 translational framshifting sites were searched for using the tRNAscan-SE program [11] and Programmed Frameshift Finder server [12]. Protein molecular weight and theoretical pI were determined using EXPASY-ProtParam [13]. Pairwise alignments of homologues proteins were conducted with the GeneStream Align algorithm [14]. Prokaryotic promoters were identified using the BGDP prediction program [15]. Potential conserved intergenic motifs were scanned with MEME/MAST [16] and PHIRE [17] to identify phage-specific regulatory elements. Putative factor-independent terminators were identified with Transterm [18] and their structure stability determined with MFold (dG< -10 kcal/mol) [19].

**Nucleotide sequence accession number.** The φ15 phage genome has been deposited in GenBank under the accession no. FR823298.

**Analyis of structural phage proteins.** Structural phage proteins were extracted from a 10^11^ purified phage suspension using a methanol/chloroform extraction (1:1:0.75, v/v/v). The protein pellet was resuspended in an SDS-PAGE loading buffer [20], boiled for 10 min and loaded onto a standard 12% polyacrylamide gel [21]. The whole lane was cut into slices which were analyzed by ESI-MS/MS as described earlier [22].

**Results**

**Genome organization and analysis**.

Genome sequencing of φ15 revealed a typical T7-like genome, comprising 39,562 bp and bracketed by 264 bp direct terminal repeats (DTR). The genome contains fifty predicted ORFs, all orientated in the same direction and leaving only 6.3% of the genome noncoding. It can be functionally divided into three regions involved in (i) host conversion, (ii) nucleotide metabolism and DNA replication, and (iii) morphogenesis and host cell lysis (Figure S4 and Table S1). With a overall G+C average of 58.2%, the highest of all ‘T7-like viruses’, φ15 approaches the high G+C content (61%) of its host. No tRNA genes were predicted, as expected for a member of the ‘T7-like viruses’.

The entire φ15 genome has 55.5% overall DNA similarity to its closest T7-homolog, the *P. putida* phage gh-1 [23] and shares 34 out of 50 φ15 ORFs with this phage. While 28 ORFs are typical for ‘T7-like viruses’, six ORFs are unique to φ15 and gh-1 and probably result from adaptation to the *P. putida* host. Five of these genes are encoded in the DNA replication region, while the particle structure genes are highly conserved. Fifteen other ORFs (except *φ15/16*), unique for φ15 and located in the early and middle region, probably represent a further adaptation to a particular group of *P. putida* strains.

**Region of host conversion.** Unlike gh-1, φ15 contains, like all other ‘T7-like viruses’, eight ORFs between its left direct terminal repeat and gene *1*, the RNA-polymerase (RNAP). However, φ15 appears to lack genes *0.3* and *0.7*. Gp0.3 (Ocr) is necessary for inhibition of Type I restriction endonucleases [24]. Gp0.7, in combination with Gp2, is necessary for shutting off of the host RNAP [25] and as a protein kinase stimulates translation of phage late mRNAs [26]. Kovalyova and Kropinski [23] indicated that Gp2 alone can inhibit the action of *P. aeruginosa* but not *E. coli* RNAP and therefore probably doesn’t need Gp0.7 for host RNAP shut-off. However other proteins in φ15 taking over Gp0.3 and Gp0.7 protein kinase functions haven’t been identified yet. Of the eight ORFs in the φ15 early region, only Gpφ15/2 showed homology to another phage protein, the functionally not characterized Gp7 of *P. aeruginosa* phage LKA1, while the other seven are pure hypothetical genes.

**Region involved in nucleotide metabolism and DNA replication.** Within this region five out of the six genes are located which have only homology to gh-1. These include the functionally annotated, gene *gh-1/3B* coding for a putative deoxynucleotide monophosphate kinase and Gpgh-1/5 probably belonging to the nucleotidyl transferase superfamily. Another feature marking the close resemblance between phages φ15 and gh-1 is the absence of Gp5.5, a H-NS inhibitor as expected, as the *P. putida* genomes contain a HU but no H-NS homolog [27]. Homing endonucleases are also common among ‘T7-like viruses’. They are contained in group I introns and function in site-specific gene conversion of the group I intron by catalyzing double strand breaks [28]. Like gh-1 and φSG-JL2, φ15 completely lacks sequences coding for such homing endonucleases, which reflects a low rate of genetic exchange of these sequences wit genetic pools harbouring them during evolution.

**Morphogenesis and host cell lysis.** Within the highly conserved late region involved in morphogenesis and host cell lysis (genes *8* to *19*) almost all genes typical for the ‘T7-like viruses’ are present. Using ESI-MS/MS analysis of denaturated phage particles after fractionation on a 1D-SDS-PAGE gel, we identified fourteen structural gene products reaching sequence coverage’s up to 78.7% (Table S2). This analysis delineated the structural region from genes *6.7* to gp*19*, hereby including also gene *6.7* and *7.3*, which have in the past been assigned to the DNA replication region. Surprisingly, four other gene products, two early (Gpφ15/5 and Gp15/7) and two middle (Gpgh-1/3B and Gpgh-1/6), were identified. The reason for their presence in the structural phage particle is unknown. Gpφ15/16, the putative capsid decoration protein, was however not identified and may have fallen below the threshold for detection.

The -1 frameshift within the gene encoding for the capsid protein, Gp10, normally observed in phage T7 translation and yielding Gp10B [29] is however not present within φ15 or gh-1. No plausible frameshifting signatures were identified and SDS-PAGE analysis of the φ15 structural proteome revealed no secondary capsid protein. This confirms the observation of Condron *et al*. [30] that in the absence of the -1 frameshift in gene *10A*, T7 capsids devoid of Gp10B are as stable as the wild type. Also, three small non-essential yet conserved genes, *19.2*, *19.3* and *19.5* [31] between gene *19* and the right DTR are absent and replaced by a single, unknown gene *gh-1/11*.

All *‘*T7-like viruses’ contain a four-component lysis system: a soluble muralytic enzyme Gp3.5, an endolysin, requires access to the cell wall peptiglycan by a small membrane protein Gp17.5, a holin, and two auxiliary proteins Gp18.5 and Gp18.7, Rz and Rz1 respectively. Homologs of these four genes were identified in the genome of φ15. Holins are divided into two classes based on their number of transmembrane domains: class I and class II have three and two transmembrane domains, respectively [32]. As the holins of φYeO3-12 and T7 were predicted to have two transmembrane domains, they represented class II holins [32,33]. However Kwon *et al*. [34] noted that all ‘T7-like virus’ holins only have one transmembrane domain when using the version 2.0 of TMHMM program [35], while other class I and II holins were accurately predicted. Further analysis of all classified ‘T7-like viruses’, revealed that none of them has two transmembrane domains and as Kwon *et al.* [34] remarked, these holins could form a new class. φ15 is an exception to the ‘T7-like viruses’ as it is predicted to contain two transmembrane domains typical for the class II holins. Gene *φ15/16*, unique for φ15, encodes for a protein with a bacterial Ig-like domain (group 2) (pfam: 3.10*10^-8^; HHpred: 1.00*10^-17^) but without any close phage homolog. Such Ig-like domains are found in bacterial and phage surface proteins such as intimins, bacterial cell adhesion molecules with 2 Ig-like domains and a C-type lectin like molecule, implying that carbohydrate recognition is important for cell adhesion [36]. Based on its small size (6.2 kDa) and on its position after the capsid protein but before the tail tubular proteins A and B one can hypothesize that Gpφ15/16 probably represents a cell adhesion protein which serves as capsid decoration on the virus particle.

**Genetic elements involved in replication, packaging and maturation.** φ15 has got a DTR of 264 bp, the longest of all ‘T7-like viruses’, which contains eight two-base variants of the heptamer 5’-CCTAAAG-3’. The DTR shares 52.4% nucleotide identity with its closest T7-homolog gh-1. Only phage T3 shares a higher identity of 53.2%. The right terminal repeat (TR) contains two additional guanine residues at its right end. The left TR is immediately followed by the CJ (concatemer junction) terminator (265 to 271) where RNAP, complexed with lysozyme, pauses during maturation and packaging [37]. The conserved 7-bp sequence (5’-ATCTGTT-3’) of the CJ terminator is found in both T7-like *P. putida* phages, φ15 and gh-1, but with a T/A substitution on the last sequence position. Immediately downstream of the CJ and upstream of the right TR, φ15 only contains two and three 2-base variants of the heptamer (5’-CCTAAAG-3’) respectively, within the region of short repeat sequences.

Downstream of the CJ terminator, two host σ^70^-like promoters are found within the sequence which are used for transcription of early genes, including the phage RNAP, which recognizes phage specific promoters for transcription of genes in the middle and late genomic region. The proteobacterial consensus sequence in the -35 (TTGACA) box is completely conserved, while two deviating positions within each host promoter are identified in the -10 (TATAAT) box (Table S3) [38].

T7 phage specific RNAP promoter sequences consist of a 23-bp consensus sequence from -17 to +6, divided into three distinct elements: (i) an upstream region (-17 to -6) for promoter recognition and binding, (ii) a region for promoter opening (-4 to -1) and (iii) an initiation and elongation site (+1 to +6) (Table S3) [39-45]. Characteristic of the ‘T7-like viruses’ is the strict RNAP template specificity for its own genome. Based on homology with T7 promoters and their intergenic position we identified twelve potential phage specific RNAP promoters, whereas gh-1 has only ten. As φ15 contains eight ORFs before gene 1 encoding for the phage RNAP, two additional phage promoters (φOL and φ(φ15/*7)*) were found in this early region compared to gh-1. A predicted phage specific RNA polymerase promoter (φ1) is present immediately upstream of the RNAP, suggesting autoregulation of this gene, as observed in gh-1 and K1F. The positions of the remaining promoters is almost identical for gh-1 and φ15, except for the absence of promoter φ(φ15/*9*) in gh-1 and φ9 in phage φ15. As these genomic promoter positions are analogous to the other ‘T7-like viruses’, regulation of phage mRNA synthesis will probably occur as described for this genus.

Positions -14, -7, -6, -4 and -3 are almost invariant among all T7 specific promoter sequences studied. However, only positions -7, -6, -3, and -7, -6, -4 are conserved within the consensus sequence of φ15 and gh-1 respectively, with a T/A substitution for both phages on position -14. The consensus sequences of the phage RNAP promoters of the two *P. putida* phages are almost identical in the –6 to -17 region, which dictates RNAP template specifity (Table S3), but differs rather strong from the other ‘T7-like viruses’. This is reflected in the very similar amino-acid RNAP regions of gh-1 and φ15 involved in specific recognition and binding with the -7 to -11 promoter region, and those necessary for making of additional contacts with the -17 to -13 promoter region (Figure S5) [46,47]. These two very similar gh-1/φ15 regions differ however strongly from the well conserved RNAP regions found in the other members of the ‘T7-like viruses’ [26]. This is in contrast to the promoter opening and intiation region (-4 to +6) which is rather dissimilar for φ15 and gh-1, but for which T7 and K1F have an almost identical sequence to φ15. The T7 RNA polymerase requires a purine for initiation on position +1 with a strong preference for G, which is present in both φ15 and gh-1 consensus sequences.

Six σ-independent transcriptional terminator sequences were identified in the genome of φ15 using visual inspection (Table S4). The σ-independent terminator (T_E_) specific for the host RNAP immediately follows the ligase gene (*1.3*) in all ‘T7-like viruses’ and hereby marks the end of transcription of the early region. φ15 however has a terminator sequence after gene *φ15/5*, following the ligase gene, which could serve this function by marking the end of the early region after this gene. Two late terminators, T_late1_, typically followed by a 6-U-stretch, and T_late2_, are located at equivalent positions to their respective counterparts in the T7 genus, after genes *10* and *16* respectively. Terminator T_late2_ has however got a reasonably high ΔG of -8.5 kcal/mol. The three remaining terminators (T_early1_, T_middle_ and T_late3_) are not typically found in T7 genomes and despite the fact that they are reasonably strong (ΔG< -10kcal/mol), further transcriptional analysis will have to point out if they really serve this function.

**References**

1. Naryshkina T, Liu J, Florens L, Swanson SK, Pavlov AR, et al. (2006) *Thermus thermophilus* bacteriphage phiYS40 genome and proteomic characterization of virions. J Mol Biol 364: 667-677.
2. Lukashin AV, Borodovsky, M (1998) GeneMark.hmm: new solutions for gene finding. Nucleic Acids Res 26: 1107-1115.
3. Altschul S, Gish W, Miller W, Myers E, Lipman D (1990) Basic local alignment search tool. J Mol Biol 215: 403-410.
4. Söding J (2005) Protein homology detection by HMM-HMM comparison. Bioinform 21: 951-960.
5. Söding J, Biegert A, Lupas AN (2005) The HHpred interactive server for protein homology detection and structure prediction. Nucleic Acids Res 33: W244-W248.
6. Kelley LA, Sternberg MJE (2009). Protein structure prediction on the web: a case study using the Phyre server. Nature Prot 4: 363-371.
7. Finn R D, Mistry J, Schuster-Bockler B, Griffiths-Jones S, Hollich V, et al (2006) Pfam: clans, web tools and services. Nucleic Acids Res 34: D247-251.
8. Möller S, Croning MD, Apweiler R (2001) Evaluation of methods for the prediction of membrane spanning regions. Bioinform 17: 646-653.
9. Lupas A, Van Dyke M, Stock J (1991) Predicting coiled coils from protein sequences. Science 252: 1162-1164.
10. Bendtsen JD, Nielsen H, von Heijne G, Brunak S (2004) Improved prediction of signal peptides: SignalP 3.0. *J.* Mol Biol 340: 783-795.
11. Lowe TM, Eddy SR (1997) tRNA-scan-SE: a program for improved detection of transfer RNA genes in genomic sequence. Nucleic Acids Res 25: 955-964.
12. Xu J, Hendrix RW, Duda RL (2004) Conserved translational frameshift in dsDNA bacteriophage tail assembly gene. Molecular Cell 16: 11-21.
13. Gasteiger E, Gattiker A, Hoogland C, Ivanyi I, Appel RD, et al. (2003) ExPASy: the proteomics server for in-depth protein knowledge and analysis. Nucleic Acids Res 31: 3784-3788.
14. Pearson WR, Wood T, Zhang Z, Miller W (1997) Comparison of DNA sequences with protein sequences. Genomics 46: 24-36.
15. Reese M G (2001) Application of a time-delay neural network to promoter annotation in the *Drosophila melanogaster* genome. Comput Chem 26: 51-56.
16. Baily TL, Gribskov M (1998) Combining evidence using p-values: application to sequence homology searches. Bioinform 14: 48-54.
17. Lavigne R, Sun WD, Volckaert G (2004) PHIRE, a deterministic approach to reveal regulatory elements in bacteriophage genomes. Bioinform 20: 629-U99.
18. Dalphin ME, Stockwell PA, Tate WP, Brown CM (1999) TransTerm, the translational signal database, extended to include full coding sequenced and untranslated regions. Nucleic Acids Res 27: 293-294.
19. Zuker M (2003) Mfold web server for nucleic acid folding and hybridization prediction. Nucleic Acids Res 31: 3406-3415.
20. Moak M, Molineux IJ (2004) Peptidoglycan hydrolytic activities associated with bacteriophage virions. *Mol* Microbiol 51: 1169-1183.
21. Laemmli UK (1970) Cleavage of structural proteins during the assembly of the head of bacteriophage T4. Nature 227: 680.
22. Lavigne R, Noben JP, Hertveldt K, Ceyssens PJ, Briers Y, et al. The structural proteome of *Pseudomonas aeruginosa* bacteriophage phiKMV. Microbiol. 152: 529-534.
23. Kovalyova IV, Kropinski AM (2003) The complete genomic sequence of lytic bacteriophage gh-1 *Pseudomonas putida* – evidence for close relationship to the T7 group. Virol 311: 305-315.
24. Walkinshaw MD, Taylor P, Sturrock SS, Atanasiu C, Berge T (2002) Structure of Ocr from bacteriophage T7, a protein that mimics B-form DNA. Mol Cell 9: 187-194.
25. Hesselbach BA, Nakada D (1977) “Host shutoff” function of bacteriophage T7: involvement of T7 gene 2 and gene 0.7 in the inactivation of *Escherichia coli* RNA polymerase. J Virol 24: 736-745.
26. **Robertson ES, Aggison LA, Nicholson AW (1994) Phosphorylation of elongation factor G and ribosomal protein S6 in bacteriophage T7-infected *Escherichia coli*. Mol Microbiol 11: 1045-1057.**
27. Liu Q, Richardson CC (1993) Gene 5.5 protein of bacteriophage T7 inhibits the nucleoid protein H-NS of *Escherichia coli*. *Proc* Natl Acad Sci USA 90: 1761-1765.
28. Dujon B (1989) Group I introns as mobile genetic elements: fact and mechanistic speculations – a review. Gene 82: 91-114.
29. Dunn JJ, Studier FW (1983) Complete nucleotide sequence of bacteriophage T7 and the location of T7 genetic elements. J Mol Biol 166: 477-535.
30. Condron BG, Atkins JF, Gesteland RF (1991) Frameshifting in gene 10 of bacteriophage T7. J Bacteriol 173: 6998-7003.
31. Kim SH, Chung YB (1996) Isolation of a mutant bacteriophage T7 deleted in non essential genetic elements, gene 19.5 and m. Virol 216: 20-25.
32. Young R, Bläsi U (1995) Holins: form and function in bacteriophage lysis. FEMS Microbiol Rev 17: 191-205.
33. Pajunen MI, Kiljunen SJ, Söderholm ME-L, Skurnik M (2001) Complete genomic sequence of the lytic bacteriophage φYeO3-12 of *Yersinia enterolitica* serotype O:3. J Bacteriol 183: 1928-1937.
34. Kwon H-J, Cho S-H, Kim T-E, Won Y-J, Jeong J, et al. (2008). Characterization of a T7-like lytic bacteriophage (φSG-JL2) of *Salmonella enteric* serovar *Gallinarum* biovar *gallinarum*. Appl Environm Microbiol 74: 6970-6979.
35. Möller S, Croning MD, Apweiler R (2001) Evaluation of methods for the prediction of membrane spanning regions. Bioinform 17: 646-653.
36. Kelly G, Prasannan S, Daniell S, Fleming K, Frankel G, et al. (1999) Structure of the cell-adhesion fragment of intimin form enterophathogenic *Escherichia coli*. Nat Struct Biol 6: 313-318.
37. Zhang X, Studier FW (2004) Multiple roles of T7 RNA polymerase and T7 lysozyme during bacteriophage T7 infection. J Mol Biol 340: 707-730.
38. Missiakas D, Raina S (1998) The extracytoplasmic function sigma factors – role and regulation. Mol Microbiol 28: 1059-1066.
39. Chapman KA, Burgess RR (1987) Construction of bacteriophage T7 late promoters with point mutations and characterization by *in vitro* transcription properties. Nucl Acids Res 15: 5413-5432.
40. Chapman KA, Gunderson SI, Anello M, Wells RD, Burgess RR (1988) Bacteriophage T7 late promoters with point mutations: quantitative foot printing and *in vivo* expression. Nucl Acids Res 16: 4511-4524.
41. Raskin CA, Diaz GA, Joho K, McAllister WT (1993) Hierarchy of base-pair preference in the binding domain of the bacteriophage T7 promoter. J Mol Biol 229: 805-811.
42. Li T, Ho HH, Maslak M, Schick C, Martin CT (1996) Major groove recognition elements in the middle of the T7 RNA polymerase promoter. Biochem 35: 3722-3727.
43. Újvári A, Martin CT (1997) Identification of a minimal binding element within the T7 RNA polymerase promoter. J Mol Biol 273: 755-781.
44. Rong M, He B, McAllister WT (1998) Promoter specificity determinants of T7 RNA polymerase. Proc Natl Acad Sci 95: 515-519.
45. Imburgo D, Rong M, Ma K, McAllister WT (2000) Studies of promoter recognition and start site selection by T7 RNA polymerase using a comprehensive collection of promoter variants. Biochem 39: 10419-10430.
46. Cheetham GM, Steitz TA (1999) Structure of a transcribing T7 RNA polymerase initiation complex. Science 286: 2305-2309.
47. Cheetham GM, Steitz TA (2000) Insights into transcription: structure and function of single-subunit DNA-dependent RNA polymerase. Curr Opin Struct Biol 10:117-123.
48. Scholl D, Merril C (2005) The genome of bacteriophage K1F, a T7-like phage that has acquired the ability to replicate on K2 strains of *Escherichia coli*. J Bacteriol 187: 8499-8503.
